# Combined clinoptilolite and Fe(O)OH for efficient removal of Cu(II) and Pb(II) with enhanced solid–liquid separation

**DOI:** 10.1007/s43938-025-00075-y

**Published:** 2025-02-25

**Authors:** Jennifer N. Enemmoh, David Harbottle, Muhammad Yusuf, Timothy N. Hunter

**Affiliations:** 1 School of Chemical and Process Engineering, University of Leeds, Leeds, LS2 9JT UK; 2 Research Center for Nuclear Materials and Radioactive Waste Technologies (PRTBNLR), Research Organization for Nuclear Energy (ORTN), National Research and Innovation Agency (BRIN), South Tangerang, 15314 Indonesia; 3 Interdisciplinary Research Center for Industrial Nuclear Energy (IRC-INE), King Fahd University of Petroleum and Minerals (KFUPM), Dhahran, 31261 Kingdom of Saudi Arabia

**Keywords:** Clinoptilolite, Co-precipitation, Dewatering, Ion exchange, Water treatment, Yield stress

## Abstract

This study investigated combining fine clinoptilolite with iron hydroxide coagulant, as a cost-effective, dual-purpose flocculant for enhanced removal of Pb^2^⁺ or Cu^2^⁺ ions, along with the solid–liquid separation and physicochemical analysis. For the clinoptilolite, adsorption kinetics fitted a pseudo-second-order (PSO) rate model with higher rate constants for Pb^2+^, while equilibrium adsorption data fitted the Langmuir monolayer model, with *Q*_*max*_ similar at 18.8 mg/g for Pb^2+^ and 18.3 mg/g Cu^2+^. TEM elemental mapping of the clinoptilolite evidenced areas of K and Fe impurities, while SEM suggested a uniform distribution of aggregates comprising a clinoptilolite core with decorated FeOOH. X-ray diffraction (XRD) indicated the FeOOH phase as α-FeOOH (Goethite) with no change in structure on inclusion of adsorbed Pb^2+^. Combined clinoptilolite-FeOOH flocs were significantly larger than FeOOH only precipitates, while flocs formed from 0.5 wt% FeOOH and 1 wt% clinoptilolite produced the fastest settling rates and greatest consolidation. Compressive yield stress data also correlated with enhanced dewatering of the combined systems, due to the dense clinoptilolite acting as a weighter material. For final metals removal, combined flocs outperformed FeOOH across a broad concentration range, achieving > 98% removal for both Pb^2^⁺ or Cu^2^⁺. The greater metals removal combined with denser floc production and improved settling features highlights significantly enhanced performance above that possible from either ion exchange or precipitation alone.

**Graphical abstract:**

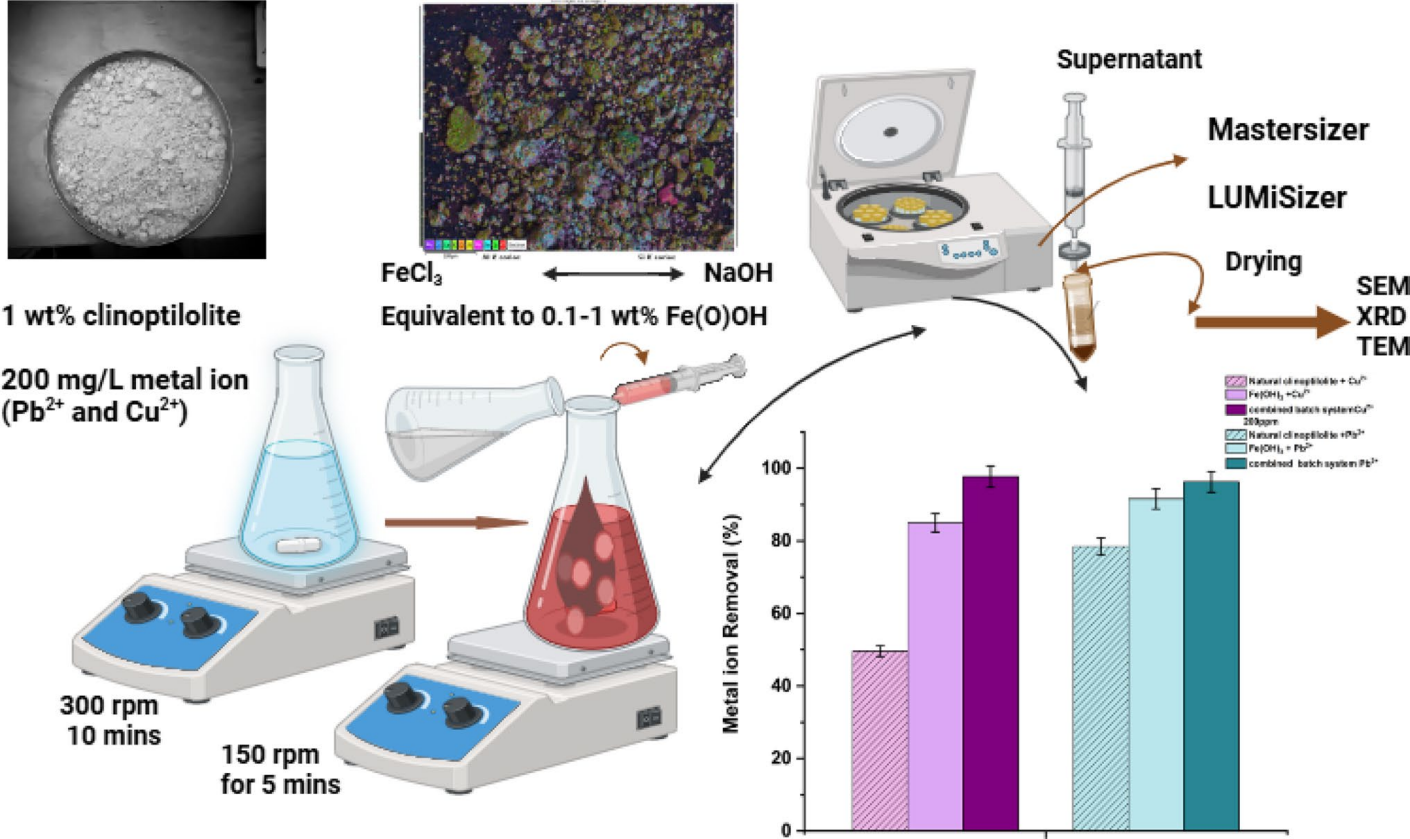

**Supplementary Information:**

The online version contains supplementary material available at 10.1007/s43938-025-00075-y.

## Introduction

Globally, lead (Pb^2^⁺) and copper (Cu^2^⁺) ions are recognized as highly toxic heavy metals, and their continuous presence in many ecosystems constitutes a severe environmental and health challenge that urgently needs to be addressed [1, 2]. Materials of lead origin, such as pipes and welding solders, have been used in various ways that exposes humans [3, 4]. Conveying water for domestic use through lead pipes, for example as practised in many developing countries, is regarded as one of the direct ways of contamination. The world health organization (WHO) and the European public health association (EUPHA) designates lead as a cumulative toxicant with a severe effect on the body system and organs. While copper is known as an essential nutrient at lower levels, excess inorganic copper intake has been shown to lead to heightened oxidant damage and increased prevalence of neurodegenerative diseases [5].

Chemical precipitation, membrane filtration, ion exchange and/or adsorption are some of the recommended methods for heavy metal removal in water treatment operations [6–10]. There has also been more recent interest in the use of various nanoadsorbents, such as bimetallic zero valent iron, MOFs or magnetic nanoparticles [11–14]. However, there are a number of challenges that are encountered with these methods as stand-alone removal protocols, such the treatment cost and complexity of downstream processing (such as for nanoparticle adsorbents) or operational issues such as fouling (e.g., in the case of membranes) [15–17]. In this view, this study investigates the combinational aspects of common ion exchange and co-precipitation methods in removing Pb^2^⁺ or Cu^2^⁺ ions from effluents to improve performance, in an operationally flexible and cost-effective way. For example, while precipitation or coagulation processes have been widely applied in treating wastewater of different origins [16, 18], the complex micro-flocs formed requires successive treatment steps such as sedimentation or filtration.

Ion exchange materials may be applied in a number of ways, with larger resins used in elution columns, while finer graded materials may be added as powder adsorbents [13, 19, 20]. Applications of naturally occurring zeolite ‘clinoptilolite’, in particular, as an efficient ion exchange material, have been reported across disciplines such as engineering, environmental science, medicine, and food and nutrition [6, 21, 22]. Nonetheless, there is a critical need to understand how to increase the efficiency of zeolites for heavy metals removal cost-effectively, especially when sourced from low-grade deposits. Generally, clinoptilolite consists of alumina silicate minerals with cation and anionic frameworks and uniquely defined cavities [23, 24]. These cavities contain microporous structures, which enable an efficient ion exchange capacity and suitability as adsorbent for heavy metals removal. Some of the beneficial aspects of clinoptilolite over other conventional ion exchange materials, such as organic chelating resins, include its availability and affordability, the release of exchangeable cations such as K⁺ and Na⁺ that are considered as non-toxic, and the stability of the inorganic clinoptilolite structure [23, 25–27]. For example, Kragovic et al. [28], studied the comparative sorption capacity of volcanic clinoptilolite-rich rock for lead and reported significantly higher sorption with Fe (iii) modified zeolite. Further, studies on the removal of heavy metals from liquid effluent using clinoptilolite as a suitable ion exchange material are well documented in the literature [2, 29–34], although, there is significant variability due to source location and influence of mineral or ion contamination that limits utilization.

It is known that milling clinoptilolite to a fine size is one proven method to increase its efficacy by exposing more surface sites [23]. This, however, leads to additional issues as it makes capture and downstream processing of the adsorbent significantly more difficult. One potential solution is to combine fine zeolite with a secondary coagulation process, involving the transformation of soluble heavy metal salt to an insoluble hydroxide that will precipitate [35]. Such a system may enhance the nature of the adsorbent to removal heavy metals more efficiently, as well as enhance downstream solid–liquid separation. Indeed, it is also well known that coagulant sludges are very difficult to process in their own right, due to the high bound water content and complex aggregated nature, requiring long sedimentation or filtration times [36–39]. Nevertheless, by combining the two processes together it may be possible to create dense composite flocs that have superior physicochemical properties to either coagulant or ion exchange alone.

While the use of ion exchange as a secondary process for clarified effluent treatment post-coagulation is more frequently utilized [40], it is much less common to combine both ion exchange and coagulation directly in a single process. Pointedly, Tonge et al. [41] explored the use of various fine mineral sediments coupled with iron hydroxide coagulation for dye removal. They found coagulation both enhanced the dye removal and improved the secondary physical separation of the flocs, due to their increased size and density. Similarly, Kivan et al. [42] recently considered the combined use of fine clinoptilolite with barium sulphate co-precipitation for the enhanced removal of strontium for nuclear effluent treatment, while Yuan et al. [43] studied the use of chitosan loaded iron oxide composite adsorbents for cadmium removal. Most directly related to the current research, Chmielewská et al., [44] examined the adsorption of fine clinoptilolite combined with iron and manganese oxides/hydroxides, which led to much improved Pb^2+^ removal, although, there was no investigation into their physical settling or filtration behavior.

Therefore, this study specifically investigates the overall performance of combined clinoptilolite-Fe(O)OH coagulation both as an affective composite adsorbent to enhance Pb^2+^ and Cu^2+^ removal, and to accelerate solid–liquid sedimentation and consolidation. In addition, kinetics and equilibrium isotherm models of the Pb^2^⁺ or Cu^2^⁺ adsorption are also determined. Removal performance of composite systems is compared to iron hydroxide only precipitates, and extensive physicochemical analysis completed on the formed flocs. In addition, the solid–liquid separation potential of the combined systems is investigated using centrifugal sedimentation testing and compressive yield stress measurements. Overall, this study seeks to understand how the clinoptilolite may act as a dual functional weighter, both enhancing metal ion removal and downstream physical separation.

## Materials and methods

### Materials

Natural clinoptilolite was supplied from Fluorochem (U.K.) as a nominal ± 7 μm powder (S25114). Sigma Aldrich supplied analytical grade heavy metal salts (purity 99%) as lead nitrate (Pb(NO₃)₂) and copper chloride (CuCl₂). Iron(III) chloride (FeCl₃) and sodium hydroxide (NaOH) of analytical grade, were also procured from Sigma Aldrich for the secondary coagulation.

### Clinoptilolite kinetic and equilibrium adsorption studies

Batch adsorption studies for Cu^2^⁺ or Pb^2^⁺ removal were investigated using stock solutions (1000 mg/L) of CuCl₂ and Pb(NO₃)₂ (diluted to either 10 or 100 mg/L) in 1 L distilled water to create a practical scenario of Cu^2^⁺ or Pb^2^⁺ loaded wastewater. Here, 0.435 g of the ion exchange adsorbent (natural clinoptilolite) was added in a 20 mL conical flask containing the prepared stock solutions of Cu^2^⁺ or Pb^2^⁺ at a constant solid to liquid ratio of 20 g/L, following the optimized procedure of Yusuf et al. [23]. The suspensions were placed on an orbital shaker and allowed to mix at 150 rpm from 15 min, 30 min, 1 h, 2 h, 4 h, 6 h, 12 h, and 24 h at ambient temperature. Afterwards, the suspensions were centrifuged at 7000 rpm for 15 min using a Heraeus Megafuge 16R (Thermo-Scientific). The supernatants were separated from the precipitate through a 20 mL syringe with a 0.3 µm filter paper. The adsorbed amount at a given time (*q*_*t*_, mg/g)) of Cu^2^⁺ or Pb^2^⁺ onto natural clinoptilolite was estimated from the concentration difference, as shown in Eq. (1); relating the initial (*C*_*o*_) and final equilibrium (*C*_*e*_) concentrations of the heavy metal ions (Cu^2^⁺ or Pb^2^⁺) in mg/L (or ppm equivalent) with the adsorbent mass (*m,* g) and volume (*V,* L) of the liquid suspension. The removal efficiency (***%***) of Cu^2^⁺ or Pb^2^⁺ from the wastewater solution is presented in Eq. (2) with *C*_*o*_ and *C*_*e*_ as previously defined.

1 $${q}_{t}=\frac{({C}_{0}-{C}_{e})}{m} V,$$

2 $$Removal\,efficiency \left(\%\right)= \frac{\left({C}_{0} - {C}_{e}\right)}{{C}_{0}} \times 100.$$

To understand the adsorption kinetics, the pseudo first order (PFO) and pseudo second-order (PSO) models were employed, as shown in Eqs. (3) and Eqs. (4)–(5).

3 $$\mathit{ln}\left[{q}_{e}-{q}_{t}\right]=-{k}_{1}t+ln{q}_{e},$$

4 $${q}_{t}= \frac{{qe}^{2}{k}_{2}t}{1+qe{k}_{2}t},$$

5 $$\frac{t}{{q}_{t}}= \frac{1}{{k}_{2}{q}_{e}^{2}}+ \frac{1}{{q}_{e} }t.$$

Here, *q*_*e*_ and *q*_*t*_ are the amounts of Cu^2^⁺, or Pb^2^⁺ adsorbed at equilibrium and time (*t*) (both in mg/g), respectively, while *k*_*1*_ is the PFO rate constant (/min) and *k₂* is the PSO rate constant (g/mg.min) [18, 23, 24].

For Cu^2^⁺ or Pb^2^⁺ equilibrium studies, 1 M stock solutions were diluted with Milli-Q^®^ (Sigma Aldrich) water to obtain varying initial concentrations. The concentration varied from 5 to 2000 mg/L and mixed with the natural clinoptilolite at a constant solid/liquid concentration of 20 g/L. An orbital shaker was used to hold the suspension for 48 h, whereupon the supernatants were separated. The data were fitted to both the Langmuir and Freundlich isotherm models to aid the understanding of the equilibrium process [26, 30]. The forms of the Langmuir isotherm are shown in Eqs. (6) and (7), respectively.

6 $${q}_{e}= \frac{{Q}_{max }{K}_{L}{C}_{e}}{1+ {{K}_{L}{C}_{e}}},$$

7 $$\frac{{C}_{e}}{{q}_{e}}= \frac{1}{{Q}_{max}{K}_{L}}+ \frac{1}{{Q}_{max}} {C}_{e}.$$

Here, *Q*_*e*_ (mg/g) represents the amounts of Cu^2^⁺ or Pb^2^⁺ adsorbed at equilibrium. *C*_*e*_ (mg/L) is the equilibrium concentration of the adsorbent. *Q*_*max*_ (mg/g) is the maximum adsorption capacity, and *K*_*L*_ (dm^3^/g) denotes the Langmuir constant. A plot of $\frac{Ce}{{Q}_{e}}$ versus *C*_*e*_ gives *Q*_*max*_ and *K*_*L*_ from the intercept and gradient, respectively. The favorability factor (*R*_*L*_) of the Cu^2^⁺ or Pb^2^⁺ adsorption onto clinoptilolite, as shown in Eq. (8), indicates the adsorption's feasibility. The lower the *R*_*L*_ value, the more favorable the adsorption process [45, 46].

8 $${R}_{L }= \frac{1}{1+{K}_{L}{C}_{0}}.$$

While the Langmuir isotherm assumes a homogenous monolayer coverage, the Freundlich isotherm explains a heterogeneous adsorption reaction [29]. The Freundlich isotherm model is as shown in Eqs. (9) and (10).

9 $${q}_{e}= {K}_{F}{C}_{e}^\frac{1}{n},$$

10 $$log{q}_{e }=log{K}_{F }+ \frac{1}{n} log{C}_{e}.$$

From Eq. (9), *K*_*F*_ (mg/g) is the Freundlich constant relating, *n* is the intensity constant of adsorption connecting the performance variance with concentration, *q*_*e*_ (mg/g), and *C*_*e*_ (mg/L) are as previously explained.

The Dubinin–Radushkevich (D–R) model was also used to evaluate the sorption energies, as given in Eq. (11) [47]. Here, the additional parameters are *β*, which relates to the sorption energy (where the free energy of adsorption, *E* = *1/(2β)*^*0.5*^ kJ/mol), *R* is the gas constant (kJ/mol.K), and *T* is the temperature (K).

11 $$\mathit{ln}{q}_{e}=ln{Q}_{max}-\beta {\varepsilon }^{2}, \text{where} \,\, \varepsilon =RTln\left(1+\frac{1}{{C}_{e}}\right).$$

The supernatant concentrations of Cu^2^⁺ or Pb^2^⁺ for all tests were measured using an atomic absorption spectrophotometer (AAS) Varian 240 fs (Agilent, Technology). All adsorption tests were conducted in triplicate and the standard deviation taken as the error.

### Production of combined ion exchange and co-precipitation composite aggregates

Batch co-precipitation combined studies were carried out employing iron(III) chloride (FeCl₃) as the coagulant to enhance the removal of Cu^2^⁺ or Pb^2^⁺ from wastewater. Initially, 4.35 g of clinoptilolite was added into an Erlenmeyer flask containing an initial concentration of 200 mg/L (Cu^2^⁺ or Pb^2^⁺) at a mixed volume of 450 mL (equivalent to 1 wt%). This suspension was mixed for 5 min at 300 rpm, after which 1 wt% of 10 mL FeCl₃ was added. The time was adjusted to 10 min at 300 rpm (rapid mixing). After the 10 min of mixing, 1 wt% of 40 mL sodium hydroxide (NaOH) was then added, and the agitation speed was reduced to 150 rpm (slow mixing) to help destabilize the colloidal particles into large flocs in a homogeneous phase. The general coagulation chemical reaction with overall stoichiometry is shown below in Eq. (12) while a schematic of the combined coagulation procedure is shown within the Supplementary Information (SI), Fig. S1. It is noted that while the overall reaction considers Fe(OH)_3_ formation, initially, precipitation results in the formation of the amorphous hydrolyzing metal salt (FeOOH) which may crystallize into a number of iron (oxy)hydroxide phases [48, 49].

12 $$Fe{Cl}_{3} + 3NaOH \to Fe{\left(OH\right)}_{3} + 3NaCl.$$

Residual Cu^2^⁺ or Pb^2^⁺ concentrations were again evaluated using atomic absorption spectrometry (AAS). Before AAS measurements, the samples were centrifuged at 7000 rpm for 10 min using a Heraeus Megafuge16R (Thermo Fisher Scientific, Cheshire, UK) and the supernatant filtered through 200 nm syringe filter [41].

Addition of 30 mL NaOH and 10 mL FeCl_3_ in the suspension should result in a 1 wt% FeOH_3_ precipitate if reacted to completion (noting there are excel levels of NaOH). The levels of the regents were in accordance with previously reported co-precipitation of liquid effluent using the same reagents [24]. To test the extent of reaction, a pH titration was completed by adding the NaOH dropwise to the dissolved FeCl_3_, with the pH changes monitored. Results are shown within the SI (Fig. S2) where there is a clear jump in pH as coprecipitation occurs, with the final pH ~ 10.5, due to the excess NaOH used to ensure complete reaction of the iron species.

Herein, samples listed as ‘*1 wt% combined*’ indicate this initial reaction combining equivalent 1 wt% clinoptilolite and 1 wt% Fe(OH)_3_. The same procedure was repeated using lower concentrations of iron chloride, resulting in various lower estimated FeOH_3_ precipitates (0.01–0.5 wt%) and using the same initial 1 wt% clinoptilolite. As a baseline, the performance of pure iron hydroxide to remove 200 mg/L of Cu^2^⁺ or Pb^2^⁺ without clinoptilolite was also investigated. Again, all tests were completed in triplicate and the standard deviation evaluated.

### X-ray diffraction (XRD), scanning electron microscopy (SEM), transmission electron microscopy (TEM) and surface area analysis

In this study, x-ray diffraction (XRD) was used to establish the crystalline form of the clinoptilolite and final phase of the precipitated iron hydroxide, as well as confirm if any changes to crystallinity were evident for the combined composite precipitates. Samples were prepared using 1 g of powder (either dried clinoptilolite, FeOOH or combined precipitate) packed into the x-ray mount. For the diffraction to occur, the spacing between the atoms must be close to the wavelength of radiation used. The measurements were taken over the 2*ϴ* angle from 10 to 60° with step size and scan speed 0.032° of 0.2 1/s, respectively. The diffraction patterns of the samples were identified and compared using the international center for diffraction data (ICDD) online database.

A High Angle Annular Dark field (HAADF) scanning Transmission electron microscope (STEM) and coupled energy dispersive X-ray spectroscopy (EDS) was used to examine the elemental surface compositions of the natural clinoptilolite. The HAADF-STEM analysis was performed on a FEI Titan^3^ Themis G2 system operating at 300 kv coupled with a 4 EDS silicon drift detector and a Gatan one view CCD. The EDS spectroscopy and elemental mapping was conducted employing the Bruker Espirit v 1.9 software. Clinoptilolite samples were firstly mixed in a 200 mg/L lead nitrate solution to visually observe Pb^2+^ adsorption, before being washed and dried. Combined coprecipitated composite samples were also investigated with scanning electron microscopy (SEM) (Hitachi SU8230) and coupled (EDS) to analyze the morphology and elemental distribution of the clinoptilolite in the precipitated flocs. Samples were firstly coated with 10 nm thickness of carbon using a Q150TE evaporative coater (Quorum) to make samples more conductive for SEM analysis.

Surface area was measured using the Brunauer–Emmett–Teller (BET) method, using a Tristar 3000 (Micromeritics). Before active measurements, each sample was degassed following a procedure previously reported [23]. Then, N_2_ gas was injected, and adsorption–desorption isotherms were recorded, where the amount of adsorbed gas per particle mass was used to calculate the specific surface area.

### Particle size distributions (PSD)

The particle size distributions of the dispersed clinoptilolite powder, combined precipitate system and Fe(OH)₃ only coagulant were investigated using a Mastersizer 3000 (Malvern Panalytical, UK). In this procedure, Milli-Q™ water was used to disperse the samples before the particle size analysis with dilutions until the required obscuration value on the software was met. The dispersed samples were pumped within the Mastersizer loop system at 1500 rpm, with measurements averaged over 10 individual readings with each measurement lasting over 10 s [41].

### Sedimentation, compressive yield stress and density measurements

Gravitational sedimentation was observed using an x-ray density profiler, LUMiReader^®^ X-Ray (L.U.M GmbH, Germany). Here, 20 mL samples of 1 wt% Fe(OH)_3_ and 1 wt% combined precipitates (containing 1 wt% clinoptilolite + 1 wt% Fe(OH)_3_) were compared to observe both sedimentation rates and bed compression. Sedimentation data was also measured under 300 rpm centrifuge using the LUMiSizer^®^ (L.U.M. GmbH, Germany) for systems containing both 0.5 and 1 wt% Fe(OH)_3_ concentrations (again comparing coagulant only to combined systems). All measurements were conducted in triplicate.

The LUMiSizer^®^ was also used to measure the compressive yield stress of sludges via differential bed height measurements under variable centrifuge (500–3000 rpm). The method was recently detailed in Kivan et al. [42], and briefly, allows estimation of the equilibrium volume fraction (*Φ*_*eq*_) based on the equilibrium bed height (*H*_*eq*_) and resulting associated yield stress for each condition (*P*_*y*_*(Φ*_*eq*_*)*) as initially established theoretically by Buscall and White [50] and Green et al. [51]. Details of the procedure are found within the SI (see Equations S1 and S2).

The density of the clinoptilolite, and dried Fe(OH)_3_ coagulant were also measured using a pycnometer, to enable estimating of volume fractions (from the mass percentages) for quantitative sedimentation and yield stress comparison. The measured density of the Fe(OH)_3_ (3.27 g/cm^3^), is in reasonable agreement with values from literature of 3.4 g/cm^3^ [52, 53], where the small difference might be related to the uncertainty of the pycnometer measurement and the formation of poorly-crystalline FeOOH species. The density of the clinoptilolite was found to be 2.15 g/cm^3^.

## Results and discussion

### Kinetic and equilibrium performance of clinoptilolite for removal of Pb^2+^ and Cu^2+^

**Fig. 1:**
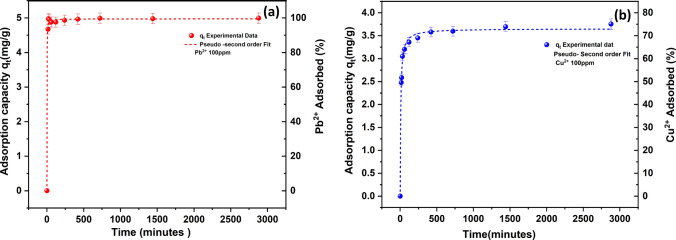
Kinetic data for (**a**) Pb^2+^ and (**b**) Cu^2+^ adsorption onto clinoptilolite at 100 mg/L dose concentrations, showing adsorption capacity (*q*_*t*_, left hand axis) and removal percentage (right hand axis), along with pseudo second order (PSO) rate fits (error bars represent ± standard deviation)

The adsorption kinetics for Pb^2+^ and Cu^2+^ are shown in Fig. 1 for a 100 mg/L dose, where the adsorbed amount is plotted against time, presented as the adsorption capacity (*q*_*t*_, left hand vertical axis) and adsorbed percentage of *C*_*0*_ (right hand vertical axis). Also shown are the calculated pseudo second order (PSO) fits (dashed lines). Adsorption data with PSO fits for the lower 10 mg/L concentration is given within the SI (Fig. S3) with pseudo first order (PFO) fits for all conditions in Fig. S4 along with a table of fitting constants (Table S1).

Observation reveals that for Pb^2^⁺, most of the adsorption occurs rapidly within the first 120 min, which is consistent with similar system in previous studies [24, 54–56]. For copper, the rate is slower, occurring within 1000 min and resulted in a lower adsorption capacity. The onset of the adsorption process indicates the presence of Pb^2^⁺ or Cu^2^⁺ concentration gradients at active surface sites of the clinoptilolite [30]. The ion exchange process between the heavy metals and the exchangeable cation present in clinoptilolite occurs during the rapid uptake period [24] and relates to the nature of the adsorbent surface layers where the cations are more easily exchangeable [57, 58]. Previous studies reported that the cavity of clinoptilolite mineral loaded with micro and macro pores would significantly improve its ion exchange capacity [24, 28, 59].

The PSO rate constant (*k*_*2*_) for Pb^2^⁺ was higher than for Cu^2^⁺ under both 10 and 100 mg/L experimental conditions (see Table 1 and Table S1). This result indicates the adsorption affinity in the order of Pb^2^⁺ > Cu^2^⁺ due to a higher mass transfer with an increase in the concentration gradient for the lead system [60]. The overall rate constants were also notably lower for the higher 100 mg/L systems, for both Pb^2+^ and Cu^2+^, which is consistent with previous literature [23], and in general is attributed to the greater exchange site competition and lower effective surface concentration gradient for higher bulk concentrations of ions. In addition, the fitness of the experimental data to the PSO kinetic model gave a high correlation coefficient (R^2^) between the 0.998 and 0.999 (Table S1), suggesting that the Pb^2^⁺ or Cu^2^⁺ adsorption mechanism onto clinoptilolite follows a chemisorption [60] process as the rate-limiting step [23, 61]. It is noted that a large number of studies have evidenced that the PSO model satisfactorily explains the adsorption process of the heavy metals onto clinoptilolite [25, 62–64]. PFO fits for 100 ppm metal ions also suggested faster kinetics for lead (*k*_*1*_-Pb^2+^ = 0.003 /min and *k*_*1*_-Cu^2+^ = 0.001/min, see Fig. S4) however, these fits suffered from relatively poor quality (R^2^ < 0.8) which is commonly observed in similar systems due to greater response sensitivity [65]. Also, the PFO model often provides closer fits in systems of primarily physisorption or electrosorption [14, 66] rather than chemisorption type interactions [67].

**Table 1:** Comparison of pseudo second order (PSO) rate constants for Pu^2+^ and Cu^2+^ ions, at varying concentrations, using zeolite-clinoptilolite minerals from different sources

Heavy metal	Adsorbent material	Concentration (mg/L)	Rate constant, *k₂* (g/mg min)	References
Pb^2^⁺	Clinoptilolite (Fluorochem, UK)	10 100	0.599 0.363	Present study
Cu^2^⁺	Clinoptilolite (Fluorochem, UK)	10 100	0.086 0.037	Present study
Pb^2^⁺	Natural zeolite (Vranjska Banja, Serbia)	4000	2.77 × 10^–4^	[59]
Pb^2^⁺	Zeolite (Australian natural deposit)	5	0.19 × 10^–2^	[21]
Cu^2^⁺	zeolite (Australian natural deposit)	5	0.27 × 10^–2^	[21]
Pb^2^⁺	Zeolite (Semnan, Iran)	300	0.0045	[74]
Cu^2^⁺	Zeolite-clinoptilolite (Brazil)	10	0.029	[68]
Pb^2^⁺	Natural clinoptilolite (Semnan, Iran)	1000	0.032	[69]
Cu^2^⁺	Synthetic clinoptilolite (China)	400	0.0005929	[75]
Pb^2^⁺	Synthetic clinoptilolite (China)	2000	0.002563	[75]
Cu^2+^	Synthetic clinoptilolite-cement composite	1000	0.00259	[76]
Pb^2+^	Synthetic clinoptilolite-cement composite	1000	0.00041	[76]

Table 1 compares the adsorption capacities for Pb^2^⁺ and Cu^2^⁺ of natural zeolites from different geographical locations to the current study. The contrasting results could be attributed to either natural variance of clinoptilolite, and the various experimental process routes and complexities of the heavy metal solution used in each study [68, 69]. The clinoptilolite in the present study has higher rate constants than several natural zeolites from different locations. It is not known where the deposit sources are for the Fluorochem clinoptilolite used (and indeed, it may not be from a single source) although, one factor that may be aiding its removal efficiency is its fine size (~ 7 µm). It has been shown specifically in previous work for cesium and strontium removal using clinoptilolite that efficiency was increased in a direct ratio to the surface area in milled samples, with the highest removal efficiency for similar fine materials [20, 23]. In general from the studies shown, the active adsorption sites of the natural clinoptilolite have a stronger affinity for Pb^2^⁺ over Cu^2^⁺ on a mass basis [57, 63, 70, 71]. Discrepancies in affinity and selectivity of clinoptilolite for heavy metal exist in literature and this disparity has been attributed to a number of factors such as initial acidity of the metallic solution and concentration [56, 72, 73].

**Fig. 2:**
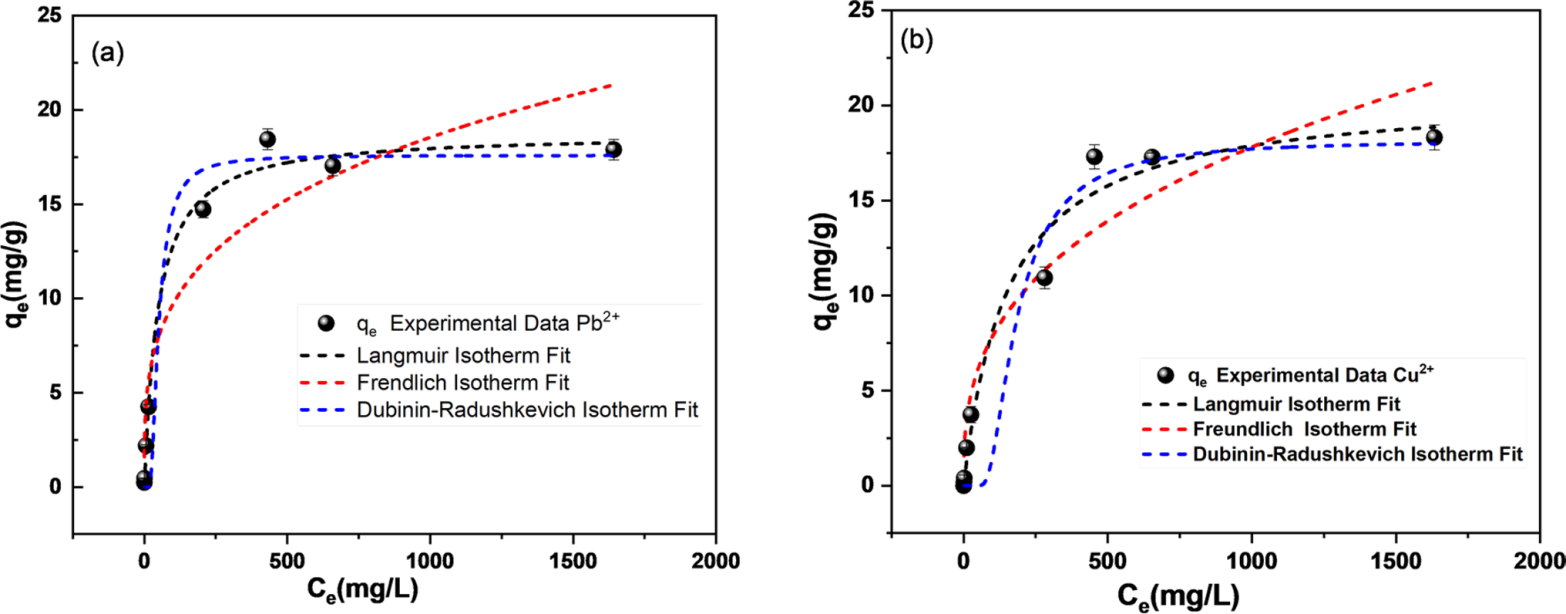
Equilibrium isotherm model fits of experimental *q*_*e*_ (mg/g) versus *C*_*e*_ (mg/L), (**a**) Pb^2^⁺ and (**b**) Cu^2^⁺ (error bars represent ± standard deviation)

Figure 2 presents the equilibrium adsorption data for Pu^2+^ and Cu^2+^, along with the Langmuir, Freundlich and D–R fits, showing the relationship between adsorption capacity (*q*_*e*_) and the adsorbate concentration at equilibrium (*C*_*e*_) [45, 77]. Fitted model parameters are given in Table 2.

**Table 2:** Equilibrium fitting constants for Pb^2+^ and Cu^2^⁺ ion adsorption onto clinoptilolite

Isotherm model	Symbol	Units	Pb^2^⁺	Cu^2^⁺
Langmuir	Q_max_	mg/g	18.72	18.32
	K_L_	dm^3^/g	0.014	0.009
	R_L_		0.4	0.5
	R^2^		0.999	0.999
Freundlich	K_F_	mg/g	1.87	1.30
	n		2.59	2.63
	R^2^		0.901	0.904
D-R	Q_max_	mmol/g	0.155	0.504
	E	kJ/mol	10.1	8.2
	R^2^		0.984	0.947

The experimental data were fitted best to the Langmuir isotherm (with R^2^ values of 0.999 compared to ~ 0.90 for the Freundlich isotherm, Table 2) suggesting a homogenous monolayer adsorption [56, 78, 79]. The R^2^ values were also > 0.95 for the D-R fits with both metals, giving confidence in the estimated adsorption energies. While adsorption at initial concentrations of 10 and 100 mg/L gave greater affinity for Pb^2+^, the higher concentrations used in equilibrium studies led to very similar *Q*_*max*_ values (noting the *Q*_*max*_ values estimated by the D-R isotherms on a molar basis is higher for Cu^2+^ than for Pb^2+^). This change suggests a relatively larger drop in performance of the clinoptilolite for Pb^2+^ adsorption at higher concentrations. Nonetheless, the higher *K*_*F*_ value for Pb^2+^ indicates that the adsorption process at low concentration is more favorable for Pb^2^⁺ than Cu^2^⁺, which is consistent with the observations from the kinetic studies. Thus, while the *Q*_*max*_ values are similar, for lower values of more relevance to effluent treatment, clinoptilolite is more favorable for Pb^2^⁺ removal.

The Langmuir constant (*K*_*L*_) as shown in Table 2 indicates the extent or rate of adsorption of the heavy metal onto clinoptilolite. Accordingly, the higher *K*_*L*_ value for Pb^2^⁺ (0.014) suggests a stronger affinity, where its adsorption energies onto clinoptilolite are higher than Cu^2^⁺ (0.009). Likewise, considering an initial concentration (*C*_*0*_ = 100 mg/L), the *R*_*L*_ value for Pb^2+^ (0.4) is slightly more favorable than for Cu^2+^ (0.5). Additionally, the adsorption free energies estimated from the D-R were higher for Pb^2+^ (10.1 kJ/mol) than Cu^2+^ (8.2 kJ/mol). Generally, energies < 8 kJ/mol are attributed to physisorption, and 8 to 16 kJ/mol to chemisorption/ion exchange [47, 80]. Accordingly, consistent with adsorption strength estimated from the Langmuir isotherm and the PSO fitting constants, both are within chemisorption range, with the lower affinity for Cu^2+^ indicating physisorption type interactions may also occur.

The *Q*_*max*_ values are also relatively consistent with previous studies on the adsorption of heavy metals using natural zeolites. Wang et al. [81], reported a *Q*_*max*_ of 12.3 mg/g in the sorption process of Cu^2^⁺ using silica oxide encapsulated natural zeolite, while Sharifipour et al. [74] reported a *Q*_*max*_ of 24.4 mg/g for Pb^2^⁺ adsorption using Iranian zeolite. Also, Li et al. [75] reported the *Q*_*max*_ values for the sorption of Cu^2^⁺ onto natural clinoptilolite from China (20.28 mg/g), natural clinoptilolite from America (22.82 mg/g), and synthetic Na-clinoptilolite from China (33.76 mg/g). Interestingly, while the PSO rate constants (*k*_*2*_) were generally higher than quoted in previous literature, the *Q*_*max*_ values are towards the lower end. This difference may be because while the final Fluorochem material has high specific surface area, it may have impurities that reduce active ion exchange adsorption sites, lowering final adsorption capacities.

**Fig. 3:**
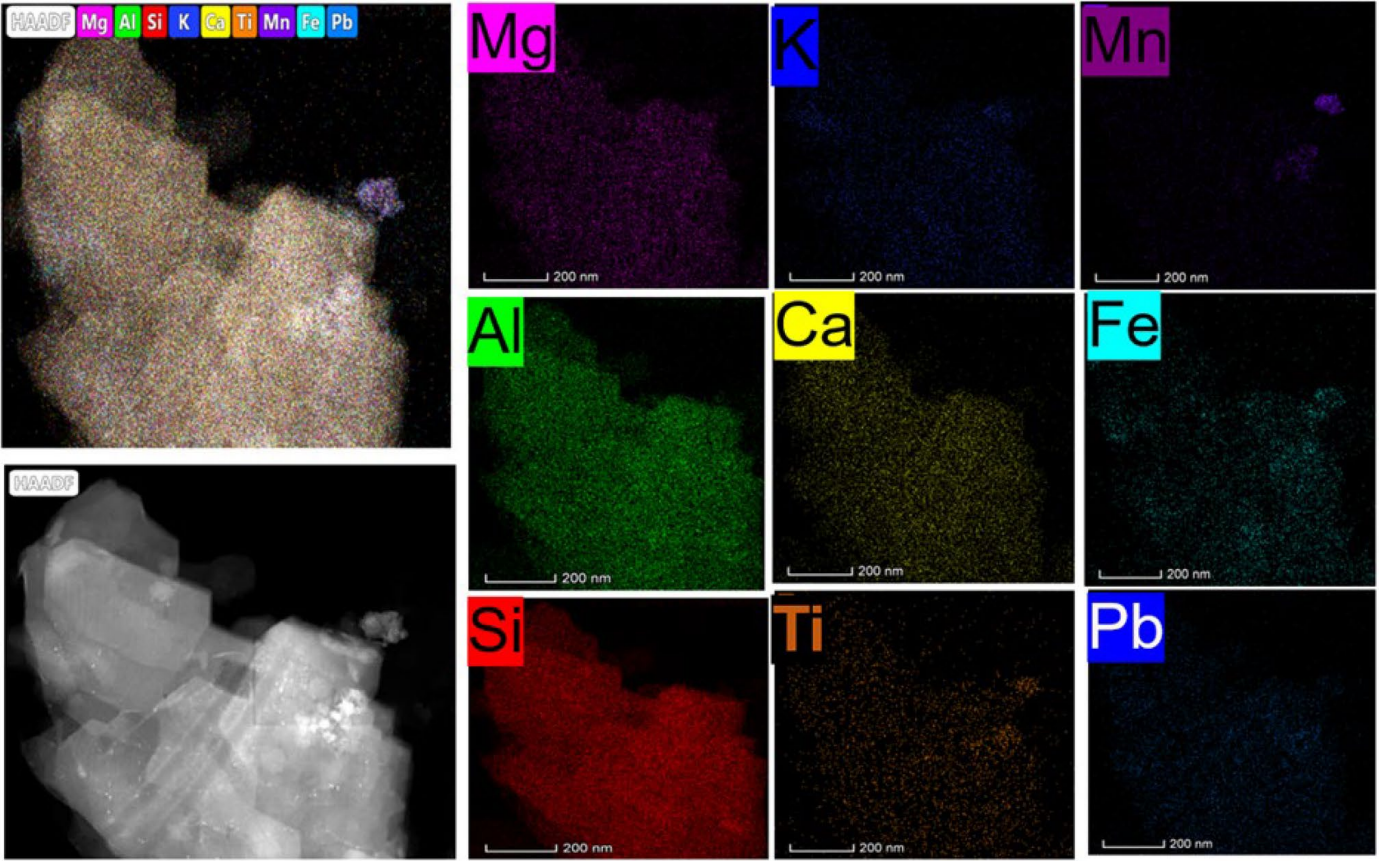
TEM-EDS elemental mapping of clinoptilolite with adsorbed Pb^2+^ and other naturally occurring contaminants

To understand the potential interference of contaminants, transmission electron microscopy (TEM) with was performed on single particles of natural clinoptilolite with pre-adsorbed Pb^2^⁺ solution (10 mg/L) along with elemental mappings used to observe the exchangeable cations. Color maps are shown in Fig. 3. It is firstly noted that the present aluminum (Al) and silicon (Si) is from the aluminosilicate clinoptilolite itself [23, 24, 72]. In terms of exchangeable ions, Pavelić et al. [72] reported that notable alkaline ions present in the cavity of clinoptilolite, which may be released during the ion exchange process due to the negatively charged surface, include Na⁺, Ca^2^⁺, Mg^2^⁺, and K⁺. All these elements are present consistently across the sample. The appearance of Pb^2^⁺, again consistently across the same, is indicative of successful monolayer coverage [23, 28, 59]. Of the exchangeable cations present in the sample, it is known that K^+^ has strong affinity for the clinoptilolite exchange sites and can act to block exchange of heavy metals reducing adsorption capacity [23]. Additionally, there are areas of high iron concentration, which may be present due to adsorption in mineral deposits and impact ion exchange, while a high-density manganese signal is present in one section, which is assumed to be to a mineral impurity.

### Physicochemical, sedimentation and yield stress characterization of composite clinoptilolite-coprecipitate flocs

**Fig. 4:**
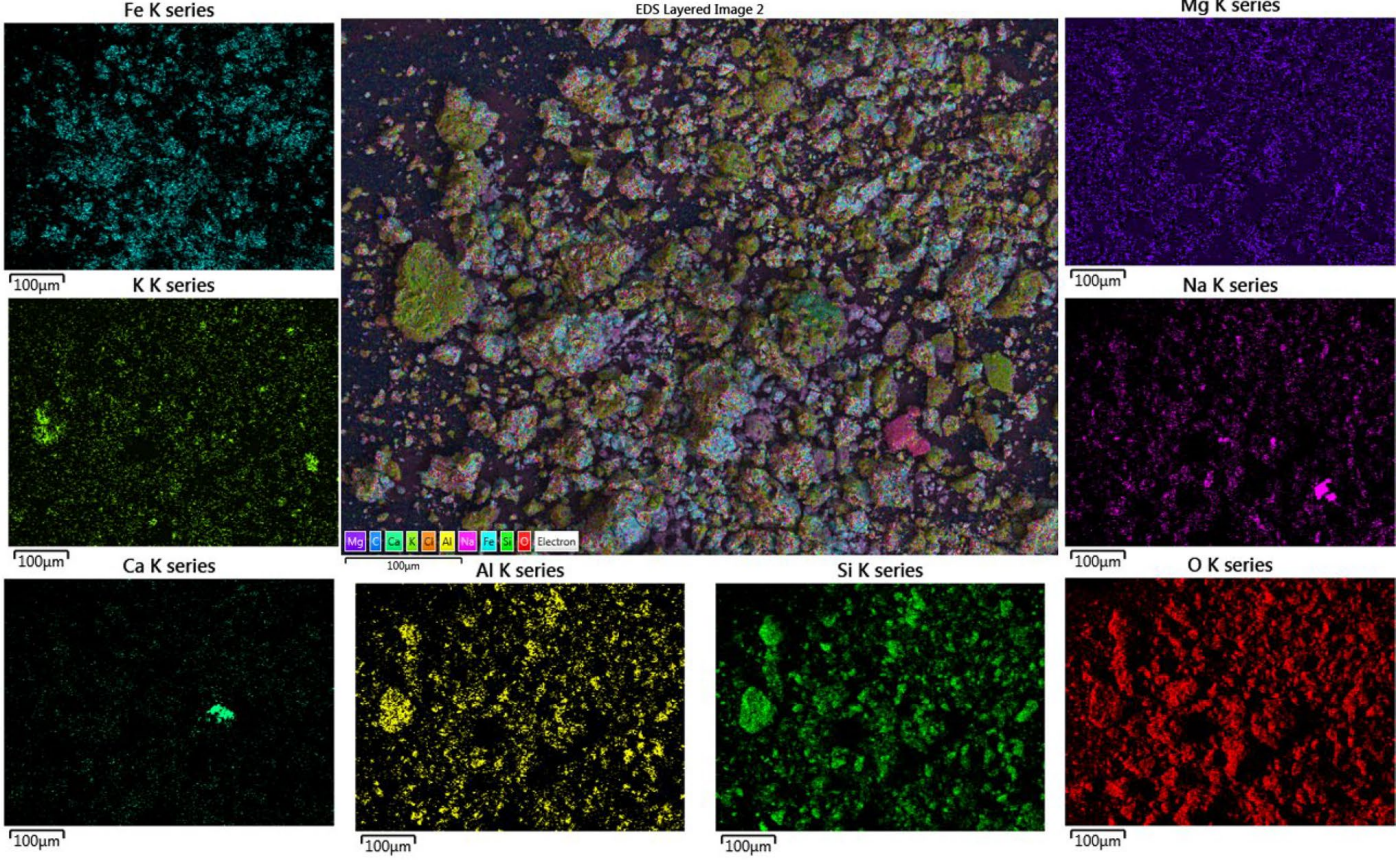
SEM–EDS of the combined clinoptilolite-Fe(OH)_3_ precipitated system

Scanning electron microscopy with elemental mapping (SEM–EDS) of the combined clinoptilolite-FeOOH flocs is given in Fig. 4, with overall elemental signal spectrum presented in the SI (See Fig. S5). What is generally evident is the relatively homogenous distribution of elements associated with the clinoptilolite (e.g., Al, Si) and those associated with the iron hydroxide (Fe). It is noted that oxygen is associated with both species, and so cannot be used to differentiate between them. On close inspection, the clinoptilolite crystals are observed as larger particles with the iron hydroxide decorated around them. Overall, there appears strong association between the clinoptilolite and hydroxide precipitate. In terms of the clinoptilolite specific exchangeable ions, there are some trace contaminants of Ca and K, which also appeared in the TEM analysis (Fig. 3) indicating the consistency in the results of the SEM–EDS with the TEM data. The Ca also appears particularly associated with one grain of clinoptilolite, which may also be due to a secondary mineral impurity.

The surface areas of the clinoptilolite and iron hydroxide were also measured by BET (see isotherms within the SI, Fig. S6). The specific surface area of clinoptilolite was 14.7 m^2^/g, and relatively consistent with previous reports of low-grade non-activated ore [23] although lower than other fine grade materials, [82] suggesting mineral contaminants may be blocking access to surface pores. Indeed, this is also consistent with isotherm being evaluated as a Type IV loop with small hysteresis, indicating nominal mesoporosity [83]. While it is known pure clinoptilolite crystal structure is generally microporous, the N_2_ gas may not be able to adsorb fully into these pores due to the inhibition of large cations [82]. The surface area of the iron hydroxide was larger (at 21.4 m^2^/g) and is consistent with its fine structure observed in the SEM. Again, its isotherm indicates Type IV with a hysteresis loop suggestive of mesoporosity, likely from the complex pore structures of bound aggregates.

**Fig. 5:**
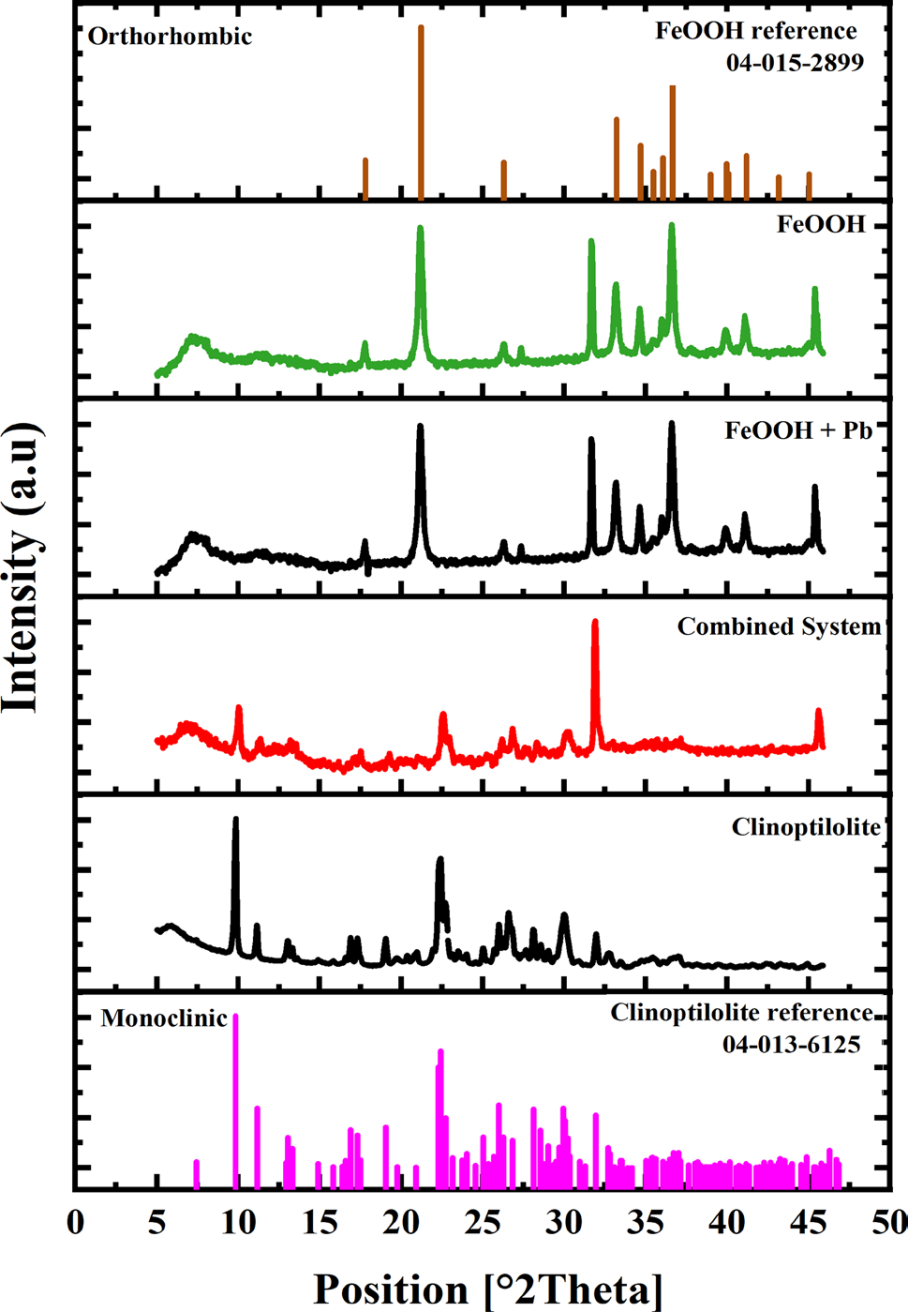
X-ray diffraction patterns of pure FeOOH, FeOOH + Pb^2^⁺ (with co-precipitated Pb^2+^), the combined system (1 wt% Fe(OH)_3_ + 1 wt% clinoptilolite), pure clinoptilolite as well as the matched references of clinoptilolite and FeOOH

The crystallinity and structural properties of the clinoptilolite, FeOOH, FeOOH with precipitated in 100 mg/L Pb^2+^ (‘FeOOH + Pb^2^⁺') and the combined composite flocs, comprising 1 wt% clinoptilolite + 1 wt% FeOH_3_ (‘Combined System’) were investigated with XRD (Fig. 5). The XRD pattern of the clinoptilolite showed a good match with the Ca-type clinoptilolite structure corresponding to the ICDD reference code of 04-013-6125 with a monoclinic crystallographic system. The cell unit parameters of the clinoptilolite (a = 17.67 Å, b = 17.95 Å and c = 7.41 Å), as evidenced by the ICDD data sheet, were consistent with those previously reported for a monoclinic Ca-type clinoptilolite [84]. The diffraction pattern of the combined system (co-precipitation coagulation of clinoptilolite with iron hydroxide) showed a decrease in intensities compared to the clinoptilolite only, although the overall pattern was still dominated by the clinoptilolite. This reduction could be attributed to the crystal size disparity and broadening owing to the decoration of the clinoptilolite with FeOOH [44].

Also presented in Fig. 5 are the X-ray diffraction patterns of iron (oxy)hydroxide (FeOOH), the main semi-crystalline iron hydroxide phase and FeOOH + Pb^2^⁺ (co-precipitated in Pb^2+^ solution). The X-ray pattern of the FeOOH is consistent with the retrieved data from ICDD with reference code of 04-015-2899 and exists as Goethite (α-FeOOH) mineral, consistent with previous literature [17]. It is also known that Goethite is more common in systems of higher pH, as in the current synthesis [85]. The crystallographic system corresponds to orthorhombic crystal. The lattice parameter of the unit cell for FeOOH were a = 4.61 Å, b = 9.95 Å, and c = 3.02 Å and angles α = β = ϒ = 90°. The XRD pattern of the FeOOH and FeOOH + Pb^2^⁺ were similar with a negligible difference in intensities and peaks, indicating that the Pb^2^⁺ caused no significant change in the crystalline structure of the FeOOH. This consistency also suggests that the presence of Pb^2^⁺ contaminants did not cause any change in the surface hydroxyl functional groups of the α-FeOOH.

**Fig. 6:**
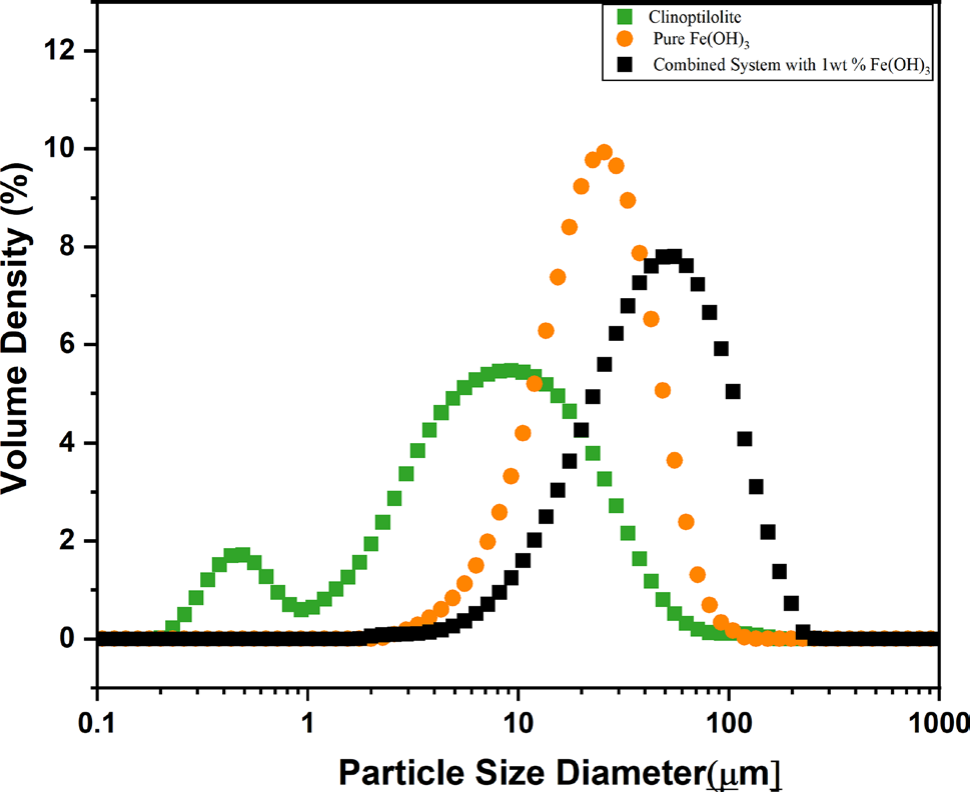
Particle size distributions (PSDs) of natural clinoptilolite, pure Fe(OH)_3_, and 1 wt% combined system with Fe(OH)₃. Presented is an average over 10 individual readings for each system

Figure 6 presents the particle size distributions of natural clinoptilolite, pure Fe(OH)_3_, and 1 wt% of the combined system (1 wt% clinoptilolite + 1 wt% FeOH_3_). The natural clinoptilolite is measured as a fine particle distribution in line with manufacturer estimates and evidence from SEM (Fig. 4). It is noted as bimodal, owing to likely production of fragments from shear in production. Such fine particles also cannot generate appreciable settling rates and might pose a major dewatering challenge. Therefore, combining the coagulant and ion exchange material allows the formation of an enlarged precipitates that subsequently should lead to easier separation. This is clearly evident in Fig. 6, with the combined system sizes significantly larger than either the clinoptilolite or the pure Fe(OH)_3_ agglomerated precipitates. The size of flocs produced with smaller 0.1 wt% and 0.5 wt% Fe(OH)_3_ were also studied (see SI, Fig. S7), where it was evident that using a lower concentration of hydroxide led to noticeably smaller overall floc sizes. As such, one may consider the 1 wt% combined system to offer potentially the greatest advantages for sedimentation acceleration. Nevertheless, it is important to emphasize that the sedimentation velocity of coagulant flocs depends not only on the floc size, but on the overall density of the floc (and its fractal dimension), viscosity of the solution, pH and other conditions owing to the differing effects on hindered settling [52, 53, 86, 87].

A previous study by Tong et al. [41], investigated various fine minerals as bifunctional weighter materials for dye removal after coprecipitating with iron hydroxide. For combined systems using fine calcium carbonate at similar concentrations, generated flocs were similar (mean sizes ~ 100 µm) which resulted in settling rates > 5 × those found with iron hydroxide only. The increase in settling rate was attributed not only to the increase in size, but importantly, to the addition of a dense adsorbent into the porous structure of the hydroxide flocs.

**Fig. 7:**
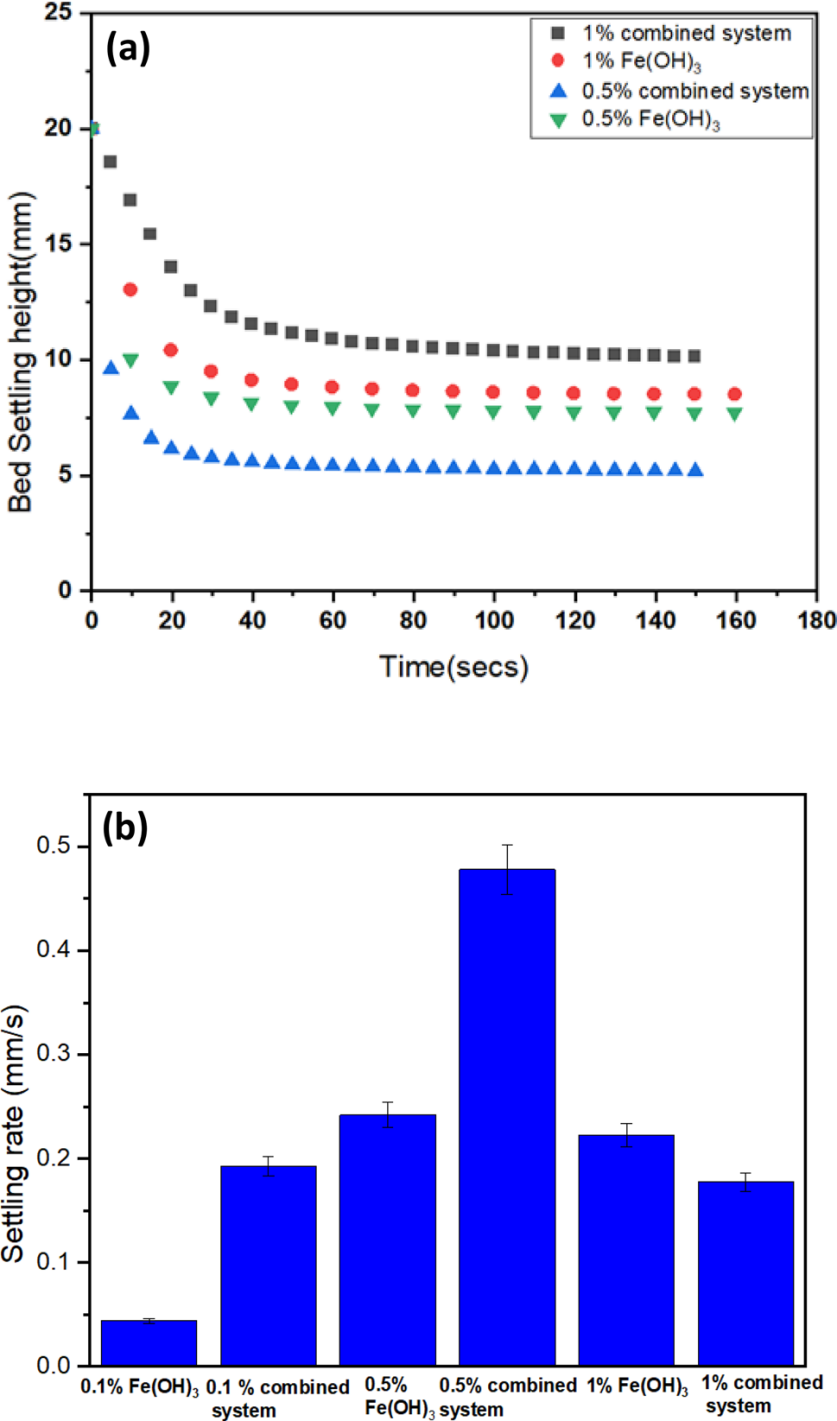
Settling of Fe(OH)_3_ precipitated flocs, along with composite coprecipitates combining 1 wt% clinoptilolite (‘Combined system’), with (**a**) example interface versus time and (**b**) initial zonal settling rates. Sedimentation generated under centrifuge at 300 rpm (error bars represent ± standard deviation)

Therefore, to confirm the interaction of the ion exchange as a weighter material in this case, the sedimentation of combined systems (1 wt% clinoptilolite + Fe(OH)_3_) and pure Fe(OH)_3_ were studied across a range of hydroxide concentrations (0.1, 0.5 and 1 wt%). Results for centrifugal settling tests conducted at 300 rpm are presented in Fig. 7 (showing the interface verse time in (a) for 0.5 and 1 wt% Fe(OH)_3_ and extracted linear zonal settling rates in (b)). Results for 0.1 and 1 wt% Fe(OH)_3_ under Earth gravity are given within the SI (Fig. S8).

There are two important trends observed. Firstly, while the particle sizes were markedly larger for 1 wt% Fe(OH)_3_ combined precipitated (Fig. 6) the settling rates are actually greater for the 0.5 wt% Fe(OH)_3_ combined system. Indeed, for the 1 wt% Fe(OH)_3_ systems (both pure and combined with ion exchange) the setting rates under centrifuge were relatively similar. This case is also true for the 1 wt% systems under Earth gravity (SI, Fig S5) where, in fact, the Fe(OH)_3_ only flocs settle slightly faster. Thus, while not producing the largest flocs, the 0.5 wt% Fe(OH)_3_ combined precipitates appear to be significantly denser with the weighted clinoptilolite material occupying a larger relative volume fraction. The likely cause of the reduced settling rates at 1 wt% Fe(OH)_3_ systems is that, while larger, the flocs produced are more open with greater water entrainment, causing an increase in the relative hindered settling as the flocs occupy a larger volume for a given solids mass [88, 89].

The influence of floc density and size on sedimentation is also observed when the level of consolidation is considered (from the final bed heights). For the 1 wt% Fe(OH)_3_ systems, the final bed heights were higher than pure 1 wt% Fe(OH)_3_ under Earth gravity and 300 rpm (although the height difference was reduced under 300 rpm). This trend would perhaps be expected, as the combined systems contained an additional 1 wt% of material, so the mass percentage of the suspension was doubled. Still, the level of consolidation was still greater for the combined system, leading to a final bed volume fraction of 0.014 versus 0.006 for pure Fe(OH)_3_ under Earth gravity (see inset Fig. S8) when converted using density values from the pycnometer. Consequently, even in this case, the weighter material aids dewatering. Nonetheless, in the case of the 0.5 wt% Fe(OH)_3_ combine system, the level of consolidation was considerably greater with the final bed heights actually being below that of pure Fe(OH)_3_ despite the addition of the 1 wt% clinoptilolite (Fig. 7a). Again, this suggests substantially greater density of the combined flocs under this concentration range that allows for easier network rearrangement and greater dewatering than is possible with Fe(OH)_3_ only systems.

**Fig. 8:**
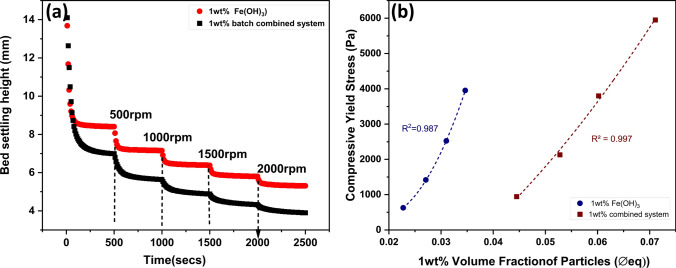
Centrifugal compressive yield stress tests for 1 wt% Fe(OH)3 and 1 wt% ‘combined system’ (1 wt% Fe(OH)_3_ + 1 wt% clinoptilolite), showing (**a**) interface versus time (rpm) and (**b**) calculated yield stress values versus equilibrium consolidated bed volume fractions

To further provide evidence of the clinoptilolite influence on consolidation and dewatering, compressive yield stress measurements were undertaken on 1 wt% pure Fe(OH)_3_ and 1 wt% ‘combined systems’ (1 wt% Fe(OH)_3_ + 1 wt% clinoptilolite), as given in Fig. 8b. Tests were conducted using the LUMiSizer^®^ from stepped increases in centrifugal speeds from 500–3000 rpm, following Kivan et al. [42], with example raw interface versus time (rpm) data shown in Fig. 8a. Data for 0.1 wt% Fe(OH)_3_ pure and combined systems are given within the SI (Fig S9).

The additional compressibility of the composite flocs are clearly evident in the yield stress data, with the exponential increase in yield stress values occurring at volume fractions considerably above those of the Fe(OH)_3_ only precipitates. This trend highlights the ability of the clinoptilolite to act as a weighter material allowing densification of the sludge bed under compression to occur and greater levels of dewatering. It is also noted that under the initial 500 rpm centrifugation speeds of the yield stress test, the equilibrium bed height of the combined system was less than the 1 wt% Fe(OH)_3_ only case. This result is in comparison to the settling test at the lower 300 rpm speed (Fig. 5) and further highlights the considerable improvement to dewatering that centrifugation gives to the combined flocs. The differences in yield stress behavior are even greater for the 0.1 wt% Fe(OH)_3_ systems (Fig. S6) although the very low hydroxide concentration made it difficult to extract accurate bed height data for the Fe(OH)_3_ flocs.

### Pu^2+^ and Cu^2+^ removal performance of composite flocs

**Fig. 9:**
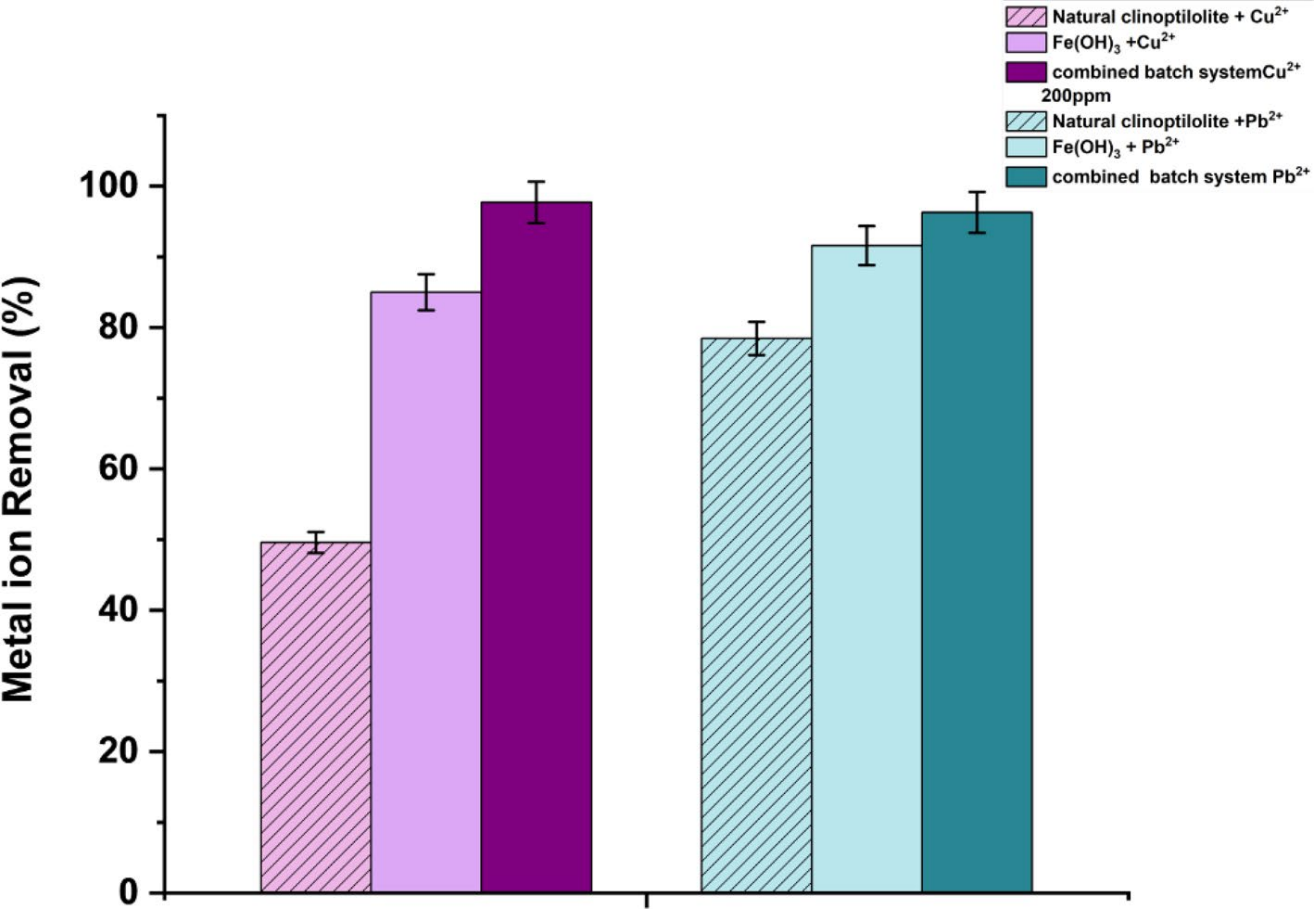
Removal of Cu^2^⁺ and Pb^2^⁺ metal ions from initial 200 mg/L solutions, for natural clinoptilolite, the combined system (with 1 wt% clinoptilolite + 1 wt% and Fe(OH)_3_) as well as pure 1 wt% Fe(OH)_3_ coprecipitation (Error bar represent ± standard deviation of three measurements). Data for natural clinoptilolite represents removal after 15 min of contact time (error bars represent ± standard deviation)

The final removal percentages of Pb^2^⁺ or Cu^2^⁺ heavy metal ions at 200 mg/L concentrations, using the natural clinoptilolite, pure 1 wt% Fe(OH)_3_ or the combined system (with 1 wt% Fe(OH)_3_ + 1 wt% clinoptilolite) were investigated as presented in Fig. 9. For both the combined system and Fe(OH)_3_, a minimum > 95% removal of Pb^2^⁺ or Cu^2^⁺ metal ions were achieved, while the removal rates for the natural clinoptilolite only was 49% for Cu^2^⁺ and 78% for Pb^2^⁺. For the clinoptilolite only systems, removal percentages were lower than for the 100 mg/L batch tests presented earlier (Fig. 4) which is to be expected as the clinoptilolite approaches its maximum cation exchange capacity [23]. Therefore, the use of the combined coagulant system is critical to increase removal at higher contaminant concentrations, with the advantage relatively greater for the Cu^2+^ metal ions. In both cases, the most complete removal was observed for the combined systems (> 97%).

Thus, it is evident that the hydroxide coprecipitation significantly increased the performance of the clinoptilolite for both heavy metals abatement at the high initial 200 mg/L concentration, but also that the co-precipitation by itself achieved relatively high removal percentage. It is important then to consider the mechanism of removal. The complexity of the iron hydroxide co-precipitation in the coagulation of heavy metals is also well-documented in literature [85, 90]. The co-precipitation produces aggregate colloids as poorly ordered ferrihydrite nanoclusters that may crystalize into a variety of tertiary structures (such as the Goethite form confirmed with XRD) [41, 49, 90]. Lead and copper may be removed via two main pathways, through either surface speciation or incorporation into the crystal lattice via substitution reactions with Fe(III) upon precipitation. In general, surface speciation is the main mechanism with both metals, with a variety of chemical reactions possible from surface hydroxyl groups, where initial aggregation of ferrihydrite into larger clusters main aid in metals retention [85]. In the current results, it is evident that the presence of the clinoptilolite does not affect these mechanisms (noting also there was no change in crystallography observed via XRD), where heavy metal ions exchanged onto the clinoptilolite may also act as link sites for the bonding with FeOOH surface hydroxyl groups.

**Fig. 10:**
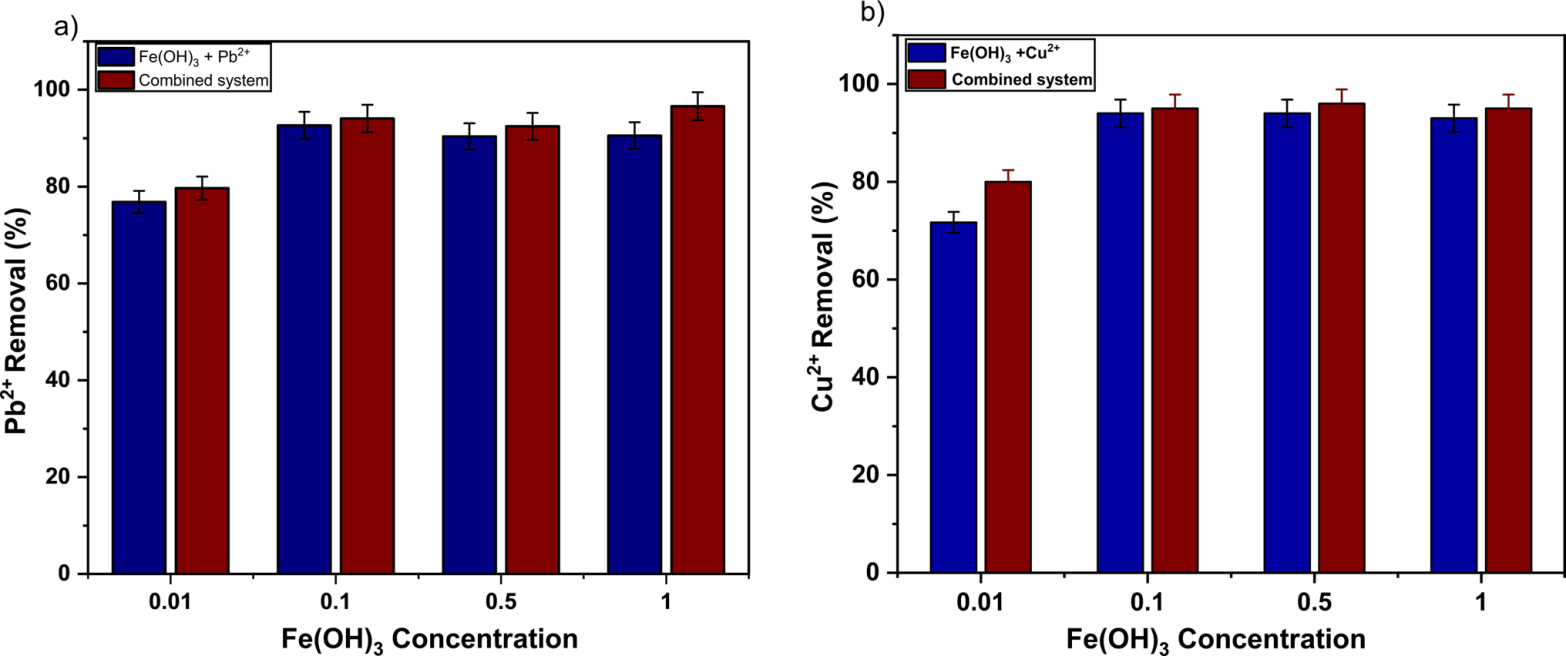
Contaminant removal performance of Fe(OH)_3_ coagulation at various concentrations with/without 1 wt% clinoptilolite; (**a**) Pb^2+^ and (**b**) Cu^2+^ ions at initial 200 mg/L concentration (error bars represent ± standard deviation)

Having established the combined performance for systems containing 1 wt% Fe(OH)₃, a full study was then completed to understand the impact of lowering the hydroxide compositions on metal removals. As it was clear from settling data (Fig. 7) that lower hydroxide composition may be preferential, it was important to determine whether that would have a detrimental effect on the metals abatement. The overall performance of the combined system and Fe(OH)_3_ for removing Pb^2+^ or Cu^2^⁺ ions from wastewater at various hydroxide concentrations is presented in Fig. 10a, b.

As observed, the combined composite removal was exceptional (at > 96%) for both metals at all hydroxide concentrations above 0.01 wt%. One very important observation is the low uptake of Cu^2+^ and Pb^2+^ at the lowest hydroxide composition (0.01 wt%) even when combined with clinoptilolite (noting, in the case of Pb^2+^, removal is slightly below that of clinoptilolite only when compared to Fig. 9). It is anticipated that this performance reduction is due to partial ion exchange between the iron(III) (when FeCl₃ is added) and lead(II) ions already adsorbed onto the clinoptilolite as they are mixed rapidly together for 10 min. This may then lead to the remobilization of some of the adsorbed Pb^2^⁺. At higher concentrations of hydroxide, any partial remobilization is not an issue as excess ions are then swept up by the precipitating hydroxide. Consequently, there is a requirement for sufficient hydroxide to speciate excess lead ions, while also enhancing the solid–liquid separation properties (noting lower concentrations of hydroxide performed poorly on sedimentation tests). Overall, results suggest that systems with 0.5 wt% Fe(OH)_3_ and clinoptilolite offer overall optimum performance, with total metal removal > 97% while achieving heightened settling rates and sludge consolidation, owing to the dual functionality of the clinoptilolite acting as both ion exchange and dense weighter material.

## Conclusions

This study examined the removal of Cu^2^⁺ or Pb^2^⁺ from heavy metal solutions using clinoptilolite as a mineral adsorbent in single-step combination with iron hydroxide co-precipitation. The focus was on understanding both how combined co-precipitation may aid total metal abatement, as well as enhance solid–liquid separation behavior. Various physicochemical analyses were also performed on the composite flocs. Batch adsorption studies with clinoptilolite were initially performed to get a clear description of the kinetic and equilibrium mechanism responsible for Cu^2^⁺ or Pb^2^⁺ removal via ion exchange. Kinetic data fitted to a pseudo second order (PSO) rate model inferring chemisorption with rate constants higher for Pb^2+^. Equilibrium data fitted to the Langmuir isotherm model, and while adsorption was more energetically preferential for Pb^2+^, maximum adsorption capacities were similar for the two metals. Elemental mapping from transmission electron microscopy (TEM), showed consistent Pb coverage and also areas of K and Fe contamination. Scanning electron microscopy (SEM) was used to analyze the structure and morphology of the combined clinoptilolite-Fe(OH)_3_ flocs, showing consisting aggregate structures consisting of a clinoptilolite core decorated with an iron hydroxide precipitate. The XRD pattern of the clinoptilolite matched with a Ca-type material with a monoclinic crystal system belonging to heulandite tecto-alumosilicate hydrate of zeolite group. The pattern of FeOOH was consistent with orthorhombic crystal and exists as a goethite (α-FeOOH) mineral, with no changes evident upon Pb^2+^ incorporation.

In terms of solid–liquid separation, composites comprising 1 wt% clinoptilolite and 0.5 wt% Fe(OH)_3_ generated both the fastest sedimentation under centrifuge, as well as higher levels of bed compression than pure Fe(OH)_3_, highlighting the role of the dense clinoptilolite acting as weighter material. This balance of composite density was critical, as aggregates made with higher hydroxide concentrations produced larger flocs that, nevertheless, produced slower settling. Compressive yield stress measurements confirmed these trends with a critical increase in yield occurring at much greater volume fractions for combined clinoptilolite-hydroxide systems than iron hydroxide precipitates. The combined systems also produced higher levels of metals removal with > 98% of Pb^2+^ or Cu^2+^ removal being achieved, greater than either clinoptilolite or iron hydroxide alone.

The obtained results overall showed that the use of natural zeolites as dual function weighers could serve as cost-effective materials to enhance Pb^2^⁺ or Cu^2^⁺ removal as well as improve sludge separation, and there are several key advantages to the combined system for industrial use. Firstly, the use of hydroxide coagulation critically improved the performance of natural clinoptilolite in removing very high concentrations of heavy metals. Secondly, while coprecipitation itself offers reasonable metals abatement, the complex open aggregate structures resulted in poor sedimentation and consolidation. By incorporating the clinoptilolite into the aggregate core, densification can be simply achieved, resulting in much lower downstream separation issues. Additionally, the one-step process is simple and could be easily retrofitted into many current water treatment facilities with the powdered clinoptilolite being added before current coagulation operations. Therefore, these composite systems could be utilized as a solution to effluent contamination in many developing and developed nations.

## Supplementary Information

Below is the link to the electronic supplementary material.

Supplementary file1 (PDF 717 KB)

## Data Availability

Data will be made publically available in the Leeds University Institutional White Rose Repository at 10.5518/1627.
