# Supplementary material for: Combined clinoptilolite and Fe(O)OH for efficient removal of Cu(II) and Pb(II) with enhanced solid–liquid separation
Source: Discov Chem Eng. 2025 Feb 25;5(1):3. doi: 10.1007/s43938-025-00075-y (PMC11861020; doi:10.1007/s43938-025-00075-y)
Supplement: Supplementary file 1 — Supplementary file1 (PDF 717 KB) [file 43938_2025_75_MOESM1_ESM.pdf]

Supplementary Information (SI)

Combined clinoptilolite and Fe(O)OH for efficient removal of Cu(II) and Pb(II) with enhanced solid-liquid separation

Jennifer N. Enemmoh[1*], David Harbottle[1], Muhammad Yusuf[2,3], Timothy N. Hunter[1*]

[1]School of Chemical and Process Engineering, University of Leeds, Leeds, LS2 9JT, U.K.

[2]Research Center for Nuclear Materials and Radioactive Waste Technologies (PRTBNLR), Research Organization for Nuclear Energy (ORTN), National Research and Innovation Agency (BRIN), South Tangerang 15314, Indonesia.

[3]Interdisciplinary Research Center for Industrial Nuclear Energy (IRC-INE), King Fahd University of Petroleum and Minerals (KFUPM), Dhahran, 31261, Kingdom of Saudi Arabia

*Corresponding Authors' email: pmjne@leeds.ac.uk; t.n.hunter@leeds.ac.uk
Phone and Fax: +44 (0)113 343 2790.

Section S1

Using Equations (S1) and (S2), the compressive yield stress at equilibrium ($P_y(\O_{eq})$) and equilibrium volume fraction ($\O_{eq}$) of the investigated systems, were calculated as given below.

$$P_y(\emptyset_{eq}) \approx \Delta\rho g \emptyset_0 H_0 (1 - \frac{H_{eq}}{2L})$$

Equation S1

$$\emptyset_{eq} \approx \frac{\emptyset_0 H_0 [1 - \frac{1}{2L}\left(H_{eq} + g\frac{dH_{eq}}{dg}\right)]}{[\left(H_{eq} + g\frac{dH_{eq}}{dg}\right)\left(1 - \frac{H_{eq}}{L}\right) + \frac{H_{eq}^2}{2L}]}$$

Equation S2

Here, $P_y(\O_{eq})$ is the compressive yield stress at equilibrium height (H_{eq}), $\O_0$ is the initial volume fraction of particle dispersions, $\Delta\rho$ is the density difference between particle and liquid in g/cm^3, g is the centrifugal acceleration at the bottom of the bed in, H_0 and H_{eq} are the initial and equilibrium sediment heights respectively in mm and L is the radial distance from centrifuge centre to the bottom of the bed in mm. The evaluated compressive yield stress is calculated for each $\O_{eq}$ (the equilibrium volume fraction of particle dispersions), at each rotational speed of the Lumiszer®.

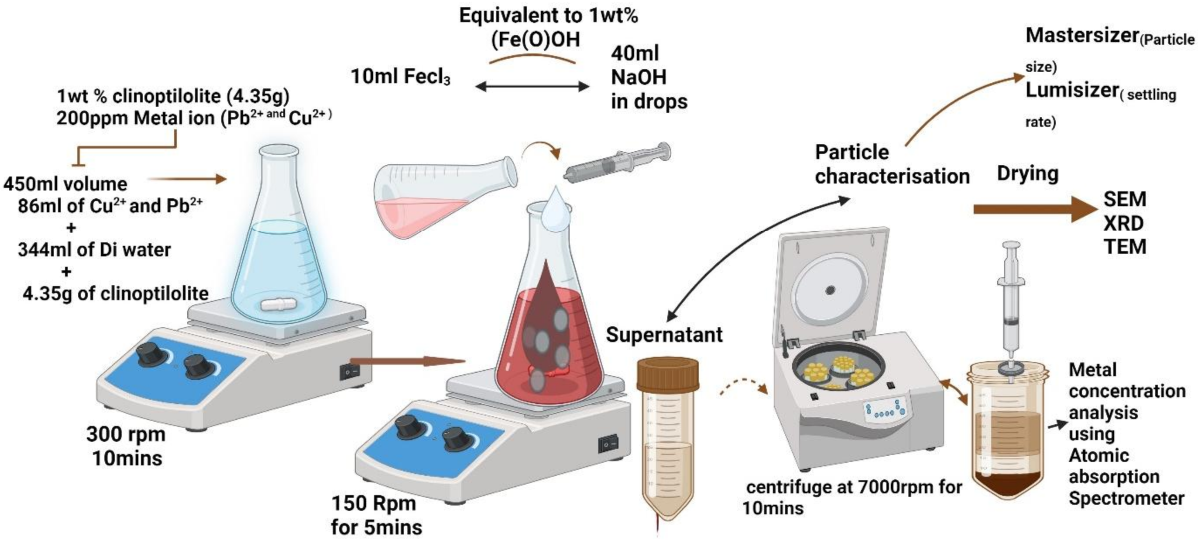

Figure S1: Schematic representation of the combined synthesis (coagulation and precipitation) and composite floc analysis.

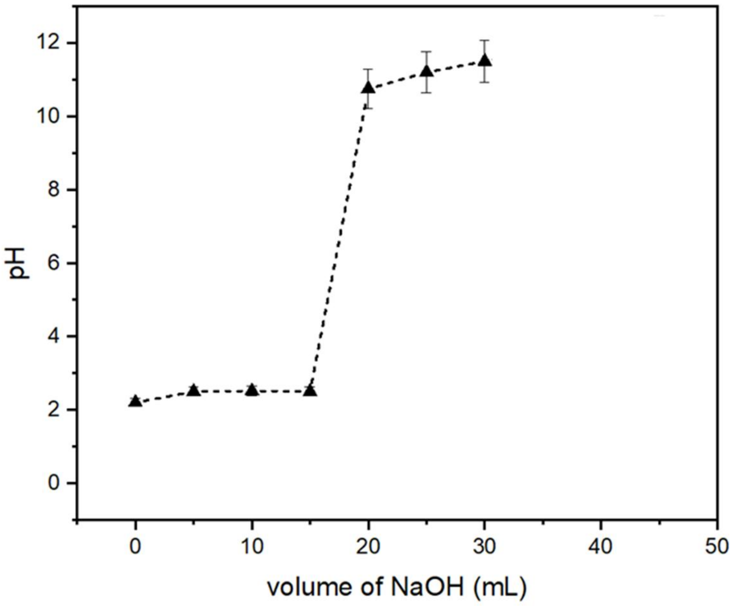

Figure S2: Titration showing change in solution pH as NaOH added dropwise to $FeCl_3$, with large increase as precipitation of Fe(O)OH occurs.

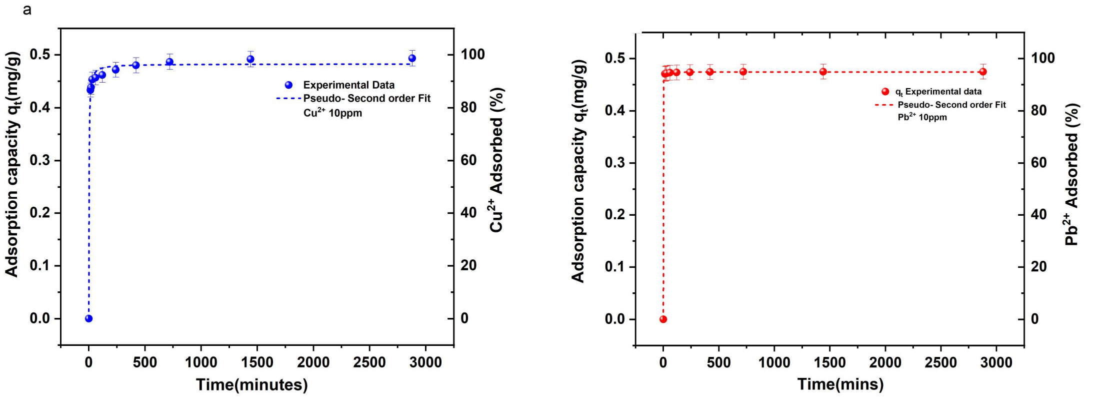

Figure S3:Kinetic clinoptilolite adsorption data, along with fitted pseudo second order (PSO) model for Pb^{2+} (left) and Cu^{2+} (right) at 10 ppm concentrations.

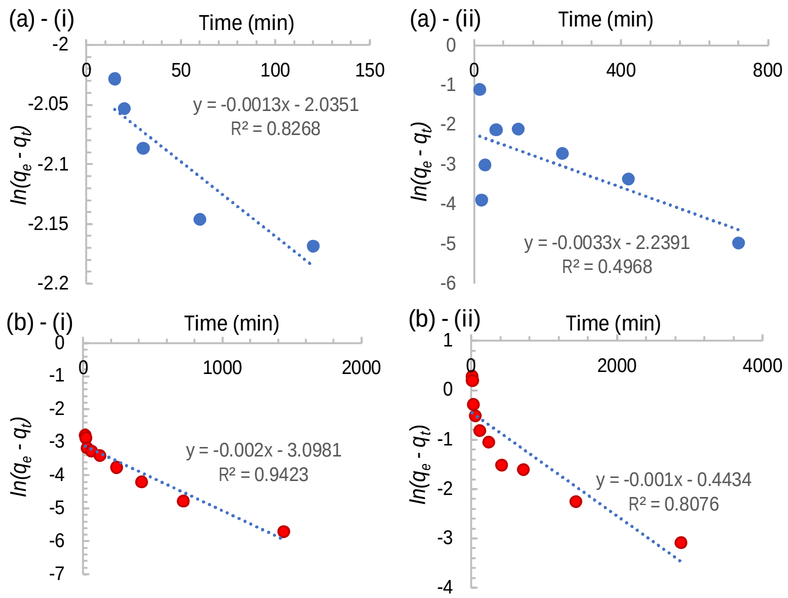

Figure S4: Linear pseudo first order (PFO) kinetic fits of clinoptilolite adsorption data, presenting (a) Pb^{2+} at 10 ppm (i) and 100 ppm (ii) and (b) Cu^{2+} at 10 mg/L (i) and 100 mg/L (ii).

Table S1: Kinetic pseudo first order (PFO) and pseudo second order (PSO) rate constants for Pb^{2+} and Cu^{2+} at 10 and 100 ppm

		Pb^{2+}		Cu^{2+}	
Parameters	Units	10 ppm	100 ppm	10 ppm	100 ppm
PFO correlation (R^2)		0.823	0.497	0.942	0.808
PFO rate constants, k_1	/min	0.0013	0.003	0.002	0.001
PSO correlation (R^2)		0.998	0.999	0.998	0.999
PSO rate constant (k_2)	g/mg.min	0.599	0.363	0.086	0.037

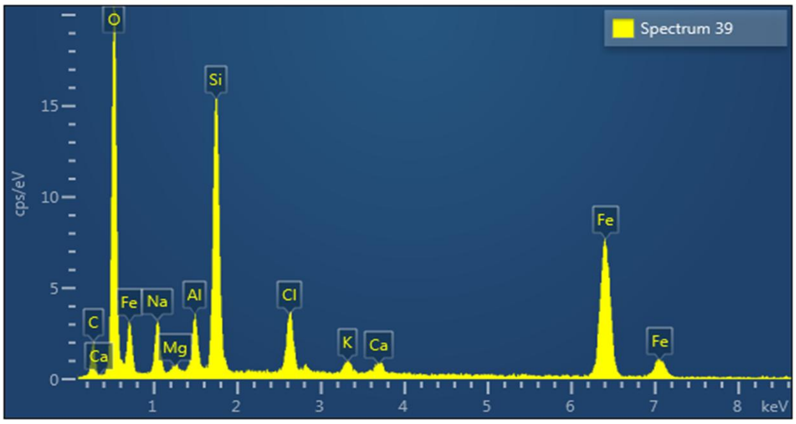

Figure S5: EDS elemental spectrum from high resolution SEM.

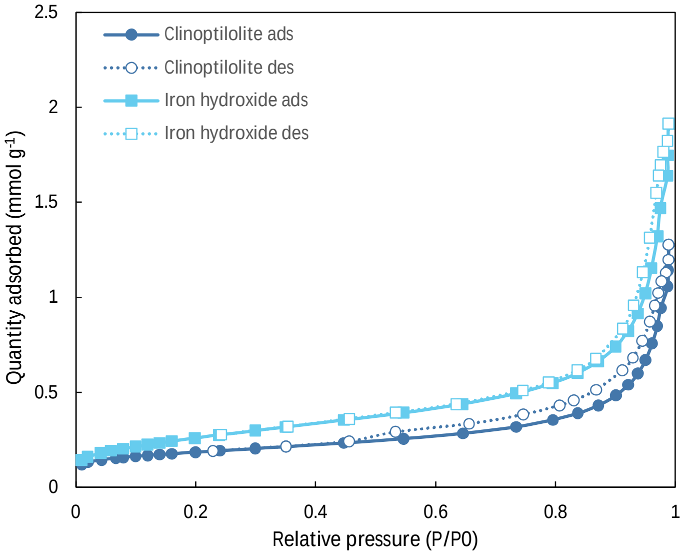

Figure S6: BET N_2 adsorption-desorption isotherms for clinoptilolite and iron hydroxide.

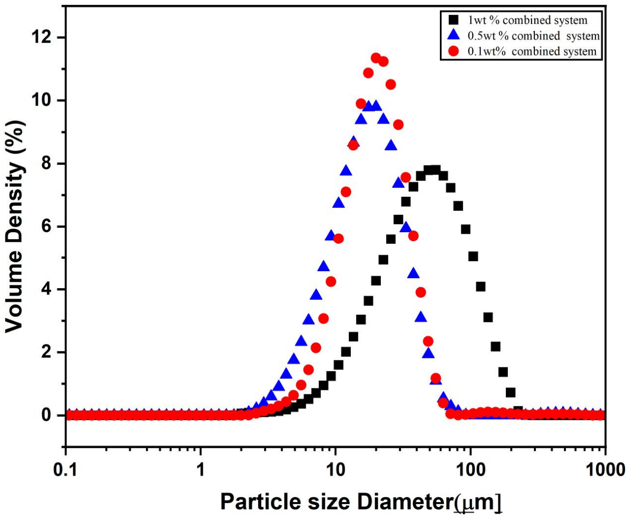

Figure S7: Particle Size Distributions (PSDs) for combined 1 wt% clinoptilolite with $Fe(OH_3)$ precipitation at 0.1 wt%, 0.5 wt% and 1 wt%.

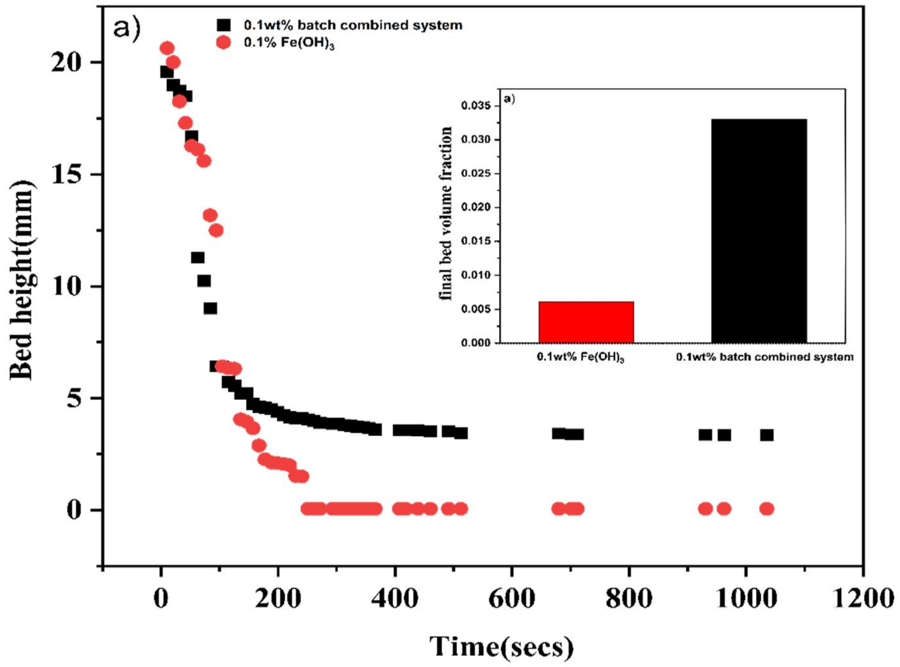

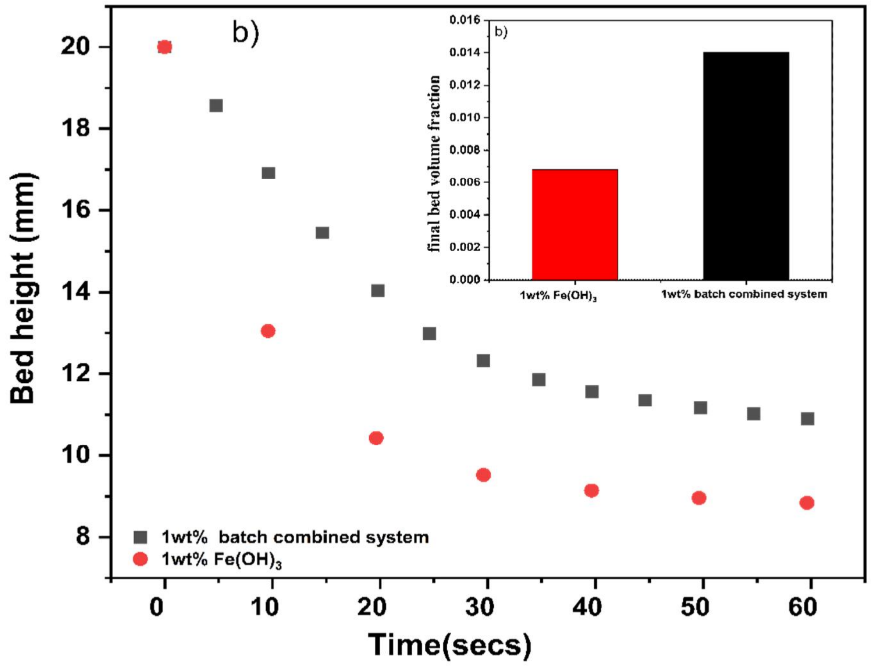

Figure S8: Earth gravity interfacial settling height versus time for pure $Fe(OH)_3$ flocs, plus 'combined systems' with additional 1 wt% clinoptilolite; (a) 0.1 wt% $Fe(OH)_3$ and (b) 1 wt% $Fe(OH)_3$.Insets in both figures show final calculated volume fractions of the consolidated beds.

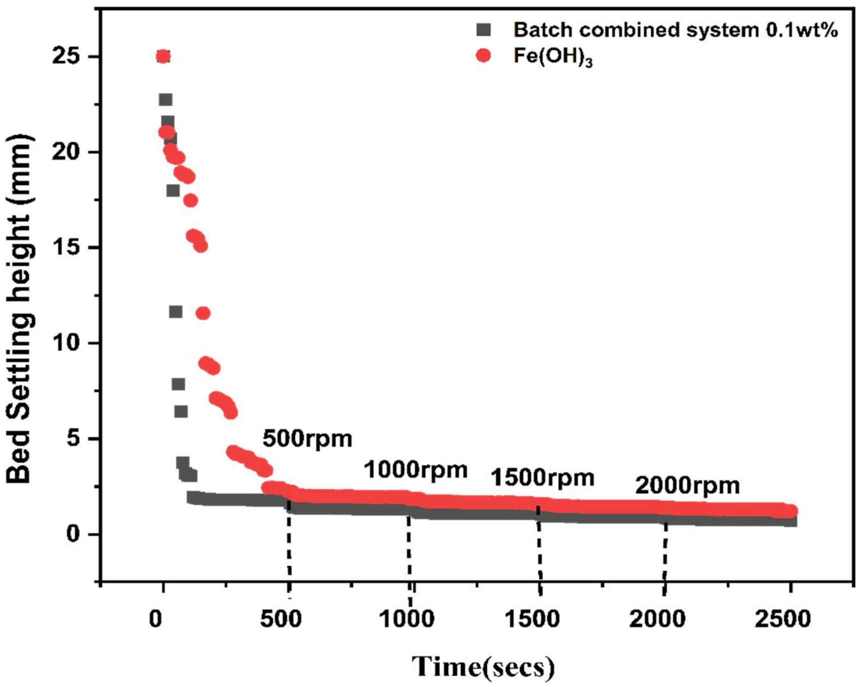

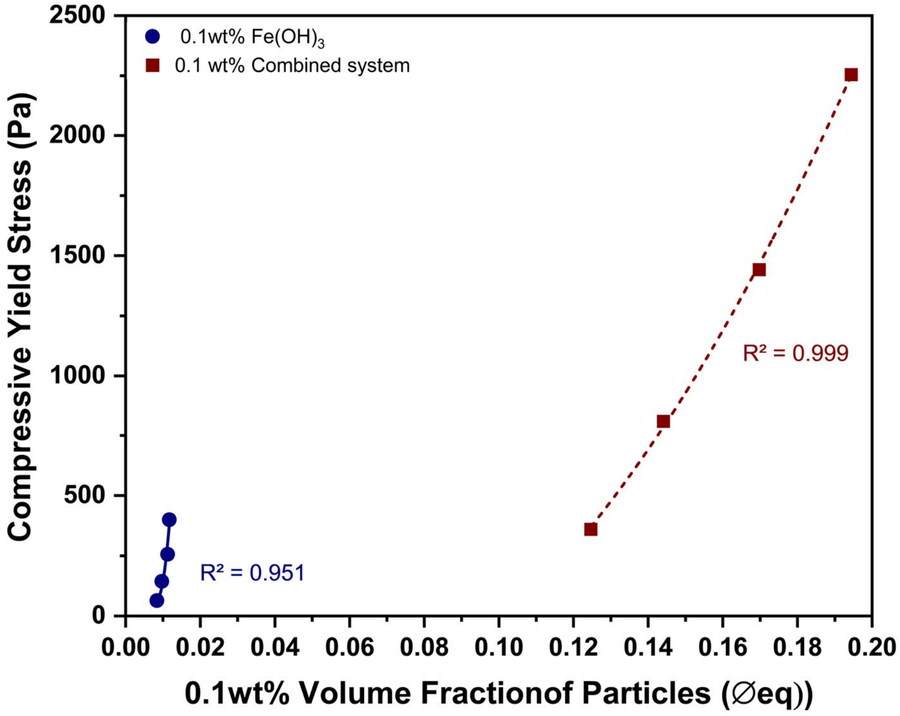

Figure S9: Stepped centrifugal interface versus rpm/time (upper) and calculated compressive yield stress (lower) for 0.1 wt% $Fe(OH)_3$ with/without 1 wt% clinoptilolite.
